# Homology Modeling, *de Novo* Design of Ligands, and Molecular Docking Identify Potential Inhibitors of *Leishmania donovani* 24-Sterol Methyltransferase

**DOI:** 10.3389/fcimb.2022.859981

**Published:** 2022-06-02

**Authors:** Patrick O. Sakyi, Emmanuel Broni, Richard K. Amewu, Whelton A. Miller, Michael D. Wilson, Samuel Kojo Kwofie

**Affiliations:** ^1^Department of Chemistry, School of Physical and Mathematical Sciences, College of Basic and Applied Sciences, University of Ghana, Accra, Ghana; ^2^Department of Chemical Sciences, School of Sciences, University of Energy and Natural Resources, Sunyani, Ghana; ^3^Department of Biomedical Engineering, School of Engineering Sciences, College of Basic & Applied Sciences, University of Ghana, Accra, Ghana; ^4^Department of Parasitology, Noguchi Memorial Institute for Medical Research (NMIMR), College of Health Sciences (CHS), University of Ghana, Accra, Ghana; ^5^Department of Medicine, Loyola University Medical Center, Maywood, IL, United States; ^6^Department of Molecular Pharmacology and Neuroscience, Loyola University Medical Center, Maywood, IL, United States; ^7^Department of Chemical and Biomolecular Engineering, School of Engineering and Applied Science, University of Pennsylvania, Philadelphia, PA, United States; ^8^Department of Biochemistry, Cell and Molecular Biology, West African Centre for Cell Biology of Infectious Pathogens, College of Basic and Applied Sciences, University of Ghana, Accra, Ghana

**Keywords:** leishmaniasis, 24-sterol methyltransferase, *Leishmania donovani*, *de-novo* drug design, molecular docking, molecular dynamics simulation

## Abstract

The therapeutic challenges pertaining to leishmaniasis due to reported chemoresistance and toxicity necessitate the need to explore novel pathways to identify plausible inhibitory molecules. *Leishmania donovani* 24-sterol methyltransferase (*Ld*SMT) is vital for the synthesis of ergosterols, the main constituents of *Leishmania* cellular membranes. So far, mammals have not been shown to possess SMT or ergosterols, making the pathway a prime candidate for drug discovery. The structural model of *Ld*SMT was elucidated using homology modeling to identify potential novel 24-SMT inhibitors *via* virtual screening, scaffold hopping, and *de-novo* fragment-based design. Altogether, six potential novel inhibitors were identified with binding energies ranging from −7.0 to −8.4 kcal/mol with e-LEA3D using 22,26-azasterol and **S1**–**S4** obtained from scaffold hopping *via* the ChEMBL, DrugBank, PubChem, ChemSpider, and ZINC15 databases. These ligands showed comparable binding energy to 22,26-azasterol (−7.6 kcal/mol), the main inhibitor of *Ld*SMT. Moreover, all the compounds had plausible ligand efficiency-dependent lipophilicity (LELP) scores above 3. The binding mechanism identified Tyr92 to be critical for binding, and this was corroborated *via* molecular dynamics simulations and molecular mechanics Poisson–Boltzmann surface area (MM-PBSA) calculations. The ligand **A1** was predicted to possess antileishmanial properties with a probability of activity (Pa) of 0.362 and a probability of inactivity (Pi) of 0.066, while **A5** and **A6** possessed dermatological properties with Pa values of 0.205 and 0.249 and Pi values of 0.162 and 0.120, respectively. Structural similarity search *via* DrugBank identified vabicaserin, daledalin, zanapezil, imipramine, and cefradine with antileishmanial properties suggesting that the *de-novo* compounds could be explored as potential antileishmanial agents.

## 1 Introduction

Visceral leishmaniasis, the most debilitating form of leishmaniasis, is caused by *Leishmania donovani* and *Leishmania infantum* (Ikeogu et al., 2020). It is one of the oldest neglected tropical diseases that remain a major challenge to the global community. It is estimated to affect over 10 million people and cause up to 30,000 deaths annually (Hernández-Bojorge et al., 2020). The present chemotherapeutic options comprising pentavalent antimony, pentamidine (PTM), amphotericin B (Amp B), miltefosine (Milt), paromomycin, and liposomal Amp B suffer from numerous inefficiencies such as long treatment durations, cytotoxicity, resistance, and high cost, necessitating the urgent need for alternative therapeutic agents (Ghorbani and Farhoudi, 2018; Sakyi et al., 2021a).

Target identification and validation are pivotal for rational drug designs (Lionta et al., 2014). Contemporary strategies comprising experimental (metabolomic and transcriptomic approaches) and computational (structure- and ligand-based) approaches have led to the identification of numerous biological targets necessary for the survival of *Leishmania* parasites (Mandal et al., 2009; Mavromoustakos et al., 2011; Rinschen et al., 2019; Kwofie et al., 2020). However, the incomplete knowledge on *Leishmania* biology and the limited studies on the exact functions of sterols in intracellular organelles have hampered the exploitation of effective sterol inhibitors against leishmaniasis. While cholesterol is biosynthesized in humans, *Leishmania* and other protozoa synthesize ergosterol. Due to this difference, a number of drugs including bisphosphonates, statins, azoles, and quinuclidine have been used for leishmaniasis treatment *via* the inhibition of the ergosterol biosynthetic pathway (Sakyi et al., 2021b). Sterol methyltransferase (SMT) is an enzyme involved in ergosterol biosynthesis which is understudied partly due to the paucity of structural genomics data. This notwithstanding, investigations are ongoing to explore SMT in designing drugs against leishmaniasis due to its absence in the human host coupled with the fact that it is highly conserved among all *Leishmania* parasites (Magaraci et al., 2003; Kidane et al., 2017).

SMT belongs to the family of transferases and functions by catalyzing methyl transfer from S-adenosyl methionine onto the C24 position of the lanosterol or the cycloartenol side chain during ergosterol biosynthesis. For example, genetic ablation studies of 24-SMT orthologs involved in the sterol biosynthetic pathway have demonstrated that ergosterol, one of the widely recognized classes of lipids in the cellular membrane of protozoans, plays a significant role in plasma membrane stabilization and mitochondrion function (Mukherjee et al., 2019). Studies have demonstrated the crucial functions of SMT to *Leishmania* survival, and hence, it is considered as a plausible target for drug design (Urbina et al., 1995; Mukherjee et al., 2019; Sakyi et al., 2021a). For instance, vaccine evaluation studies against *Leishmania* 24-SMT identified 24-SMT as an essential drug target (Goto et al., 2009). *Leishmania* 24-SMT dysfunction results in the increased generation of reactive oxygen species and vesicular trafficking (Mukherjee et al., 2019). In addition, 24-alkyl sterols have been shown to be essential growth factors of *Trypanosoma cruzi* to the extent that its perturbation during ergosterol biosynthesis leads to cell cycle defects and DNA fragmentation (Urbina et al., 1995; Pérez-Moreno et al., 2012). Furthermore, RNA-seq analysis has revealed genomic instability at the locus of SMT, resulting in the promotion of amphotericin B resistance in *Leishmania* parasites (Pountain et al., 2019). Similarly, gigantol and imipramine suppressed the growth and proliferation of promastigotes and amastigotes *via* inhibition of SMT (Andrade-Neto et al., 2016; Rahman et al., 2021). Similarly, the antiproliferative effects of sterol biosynthesis inhibition on *Pneumocystis carinii* have hinted sterol methyltransferase suppressors as potential chemotherapeutic options for the treatment of *P. carinii* infections (Urbina et al., 1997). However, the recent resistance associated with 22,26-azasterol targeting SMT warrants the identification of novel inhibitors.

*In-silico* techniques in drug design are advantageous due to the reduced cost, time, and energy compared with traditional high-throughput screening (HTS) (Mak and Pichika, 2019). One of the strategies employed in the identification of lead compounds with improved efficacy in rational drug design includes scaffold hopping (Hu et al., 2017). It starts with a known active compound and ends up with new chemotypes with different core structures but with equal or improved efficacy (Hu et al., 2017). A typical example is the discovery of cyproheptadine from pheniramine, an antihistamine used to treat allergic conditions, such as hay fever or urticaria (Sun et al., 2012). Pheniramine has two aromatic rings joined to one carbon or nitrogen atom and a positive charge center. Cyproheptadine, an analog of pheniramine, has significantly improved binding affinity against the H1 receptor. This rigidified molecule with better absorption was achieved by locking both aromatic rings of pheniramine to the active conformation through ring closure and by introducing the piperidine ring to further reduce the flexibility of the molecule (Sun et al., 2012). In addition, these structural changes gave other medical benefits including cyproheptadine as a prophylaxis for migraine, pizotifen for the treatment of migraine, and azatadine as a typical potent sedating antihistamine (Sun et al., 2012). Tramadol was obtained through scaffold hopping of morphine (Sun et al., 2012). The recent interest in *de-novo* drug design compared with repurposing presents a new paradigm shift, not only in terms of time and cost but also innovation (Talevi and Bellera, 2019). The identification of leads from hits and then optimization to druggable candidates in the drug design pipeline result not only in an increase in potency and selectivity but also improved drug-like properties (Gil and Martinez, 2021). In addition, *de-novo* drug design has increased the areas explored in the chemical space of molecules leading to improvements in chemotherapeutic efficacy (Lin et al., 2020). This strategy has also been used in the design of drugs including vemurafenib, venetoclax, and dihydroorotate dehydrogenase inhibitors against malaria and aryl sulfonamide, a new aurora A kinase inhibitor (Jacquemard and Kellenberger, 2019). Despite the success achieved using the *de-novo* design, its use in the search for potential hits against *L. donovani* 24-sterol methyltransferase (*Ld*SMT) is limited.

The study sought to utilize *in-silico* approaches to predict putative inhibitors targeting *Ld*SMT. To accomplish this, the three-dimensional (3D) structure of *Ld*SMT was first elucidated *via* modeling followed by subjection of 22,26-azasterol to *de-novo* drug design. Next, molecular docking and molecular dynamics simulation studies of the complexes were undertaken to identify potential novel *Ld*SMT inhibitors. Furthermore, the biological activity and pharmacological profiles of the compounds were predicted to reinforce their lead-likeness.

## 2 Methods

**Figure 1:**
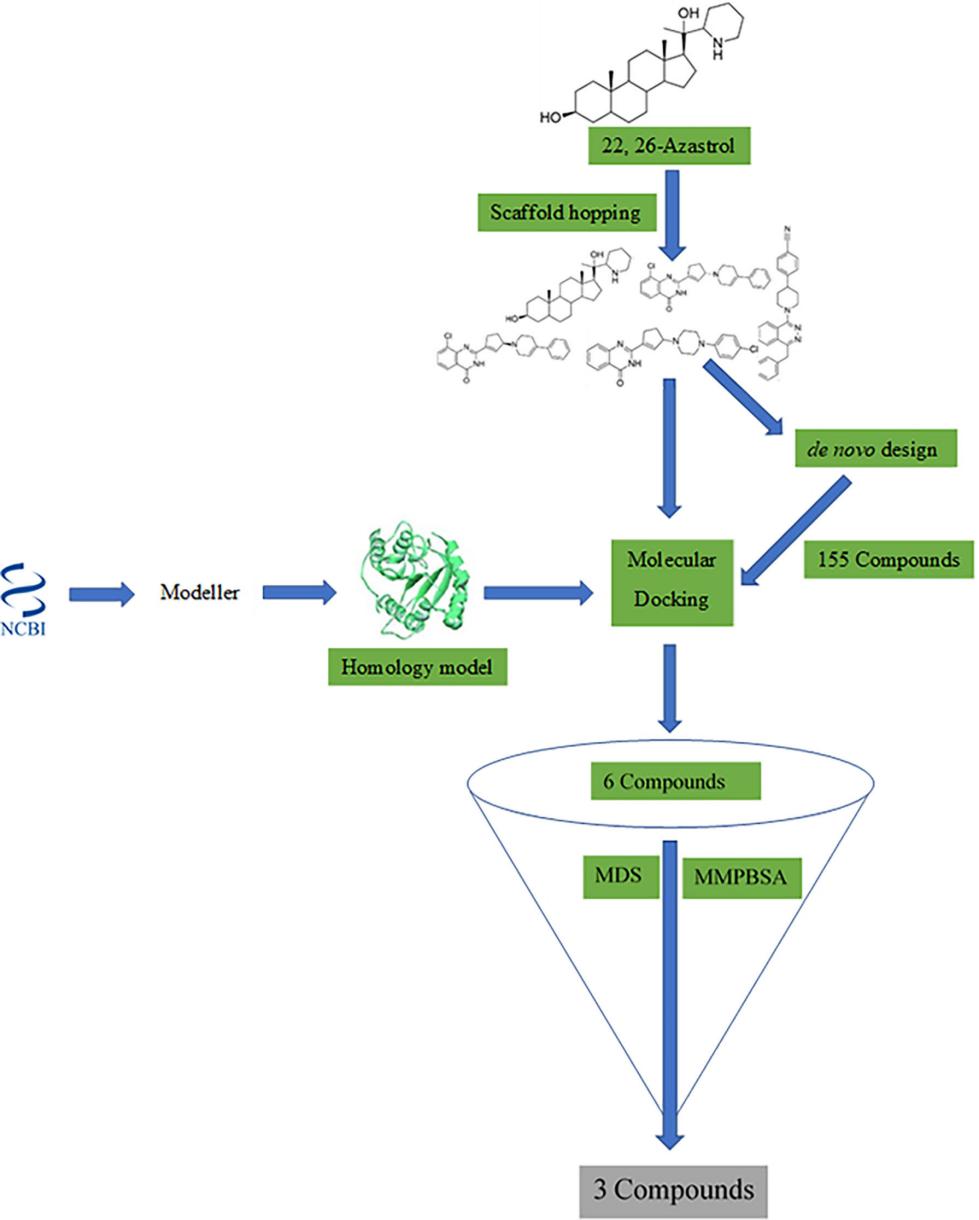
Methodology schema employed in the study for predicting antileishmanial agents.

A workflow schema detailing the stepwise techniques employed in this study is shown in **Figure 1**. The compound 22,26-azaserol was submitted to balanced rapid and unrestricted server for extensive ligand-aimed screening (BRUSELAS) (Banegas-Luna et al., 2019) to generate non-steroidal inhibitors. Meanwhile, a reasonably good structure of *Ld*SMT was modeled and validated. The non-steroidal inhibitors were virtually screened against the *Ld*SMT to identify compounds with high binding affinity to the receptor. The complexes of these compounds then served as input to the e-LEA3D (Douguet, 2010) for the generation of the novel compounds. Molecular dynamics (MD) simulation and molecular mechanics Poisson–Boltzmann surface area (MM-PBSA) were computed on the *Ld*SMT–ligand complexes to determine the molecular interactions as well as the stability during the simulation. Absorption, distribution, metabolism, excretion, and toxicity (ADMET) predictions were performed to evaluate the pharmacological profiles of the selected compounds. The inhibitory constant, ligand efficiency, ligand efficiency scale, fit quality, binding efficiency index, surface efficiency index, and ligand efficiency-dependent lipophilicity were also calculated to assess the quality parameters of the ligands in binding to the target protein. In addition, the biological activity of the selected hits was predicted using the open Bayesian machine learning technique (Lagunin et al., 2000).

### 2.1 Sequence Retrieval

The protein sequence of *Ld*SMT (SCMT1/GenBank ID: AAR92099.1) was retrieved from the National Center for Biotechnology Information (NCBI) database (Boratyn et al., 2013). To identify similar proteins as templates for building the 3D structure of *Ld*SMT, the fast-all (FASTA) format of the sequence was aligned with the homologous sequences of crystal protein structures in the Protein Data Bank (PDB) (Berman et al., 2000) using the Basic Local Alignment and Sequencing Tool (BLAST) (Burley et al., 2017).

### 2.2 *Ld*SMT Model Generation

Due to the unavailability of an experimentally elucidated 3D structure for *Ld*SMT, molecular modeling techniques were used to predict a reasonably accurate protein structure (Crentsil et al., 2020). Modeller version 10.2 (Eswar et al., 2008) was employed in building the 3D structure using three different templates: i) the 3BUS template was used to generate five models of the *Ld*SMT (Eswar et al., 2008), ii) the protein structure with PDB ID 4PNE was also used to model five different structures of the *Ld*SMT (Eswar et al., 2008), and iii) multitemplate homology modeling was employed to generate five structures of the *Ld*SMT (Eswar et al., 2008). A total of three templates comprising 4PNE, 3BUS, and 6UAK were used in the multitemplate homology modeling approach. Modeller 10.2 was employed in generating all the potential structures of the *Ld*SMT (Fiser and Šali, 2003; Eswar et al., 2008). The reasonably best model in each approach was selected based on the discrete optimized protein energy (DOPE) scores (Eswar et al., 2008).

### 2.3 Structural Validation

The structural quality and accuracy of the best models from each approach were assessed using PROCHECK (Laskowski et al., 2012) with results reported as Ramachandran plots (Anderson et al., 2005). Further validation with VERIFY 3D (Sasin and Bujnicki, 2004), ERRAT (Colovos and Yeates, 1993; Messaoudi et al., 2013), and PROVE (Sasin and Bujnicki, 2004) was also performed. The reasonably best model was selected based on all the quality assessments for the molecular docking studies. Protein Structure Analysis (ProSA) (Wiederstein and Sippl, 2007) was then used to investigate the problematic regions of the selected model.

### 2.4 Determining Binding Sites

The plausible binding sites of the selected protein were determined using the Computed Atlas of Surface Topology of proteins (CASTp) (Dundas et al., 2006; Tian et al., 2018). The predicted sites were visualized using PyMOL (PyMOL Molecular Graphics System, Version 1.5.0.4, Schrödinger, LLC, New York, USA) (Lighthall et al., 2010) and Chimera 1.16 (Pettersen et al., 2004). The predicted binding sites with relatively small areas and volumes, where no ligand could fit, were ignored (Agyapong et al., 2021).

### 2.5 Scaffold Hopping

Shape similarity searching and pharmacophore screening were undertaken using the spatial data file (sdf) format of 22,26-azasterol *via* BRUSELAS (Banegas-Luna et al., 2019). A total of 100 ligands were generated with varying degrees of similarities to the input ligand, 22,26-azasterol. All ligands devoid of the steroidal core with varying degrees of similarities were selected for binding affinity prediction using AutoDock Vina v1.2.0 (Trott and Olson, 2010).

### 2.6 Molecular Docking Studies

AutoDock Vina (Trott and Olson, 2010) was used for molecular docking. The molecular docking process was performed in two different stages. The first docking stage involved ligands obtained from the scaffold hopping process (Pathania et al., 2021), while the second involved compounds obtained from the *de-novo* design studies (Agyapong et al., 2021). Altogether, 1,448 ligands were used for the docking studies.

For the first stage, the ligands were obtained from the scaffold hopping and their structural derivatives fetched from the DrugBank (Wishart et al., 2018), PubChem (Kim et al., 2021), ZINC15 (Sterling and Irwin, 2015), and ChemSpider databases (Pence and Williams, 2010). Compounds labeled X1, X2, X3, X4, X5, X6, and X7 which showed good half-maximum inhibitory concentration (IC_50_) values against *Leishmania* parasite’s sterol methyltransferase together with amphotericin B, miltefosine, paromomycin, and 22,26-azasterol were also used.

The ligands generated from the *de-novo* design were virtually screened against the *Ld*SMT in the second stage. For both stages, the ligands and the protein were prepared using the AutoDock Tools (Morris et al., 2009) and saved in the input format of AutoDock Vina (Trott and Olson, 2010). The charge, hydrogen bond network, and histidine protonation state of the protein were assigned after pdbqt conversion. Grid box size was set to (91.445 × 73.502 × 78.352) Å^3^ with the center at (72.200, 58.009, 13.302) Å. Ligands were then screened against the *Ld*SMT protein with exhaustiveness set to default 8.

### 2.7 *De-Novo* Drug Design

The potential protein–ligand complex from the scaffold hopping was submitted to e-LEA3D (Douguet, 2010) for further *de-novo* design. The binding site radius was set to 15 Å and the final score set to 1 with the same active site coordinates as used in the molecular docking study. Conformational search was set to 10, number of generations to 30, and population size to 30 with the rest of the options left as default.

### 2.8 Characterization of the Mechanism of Binding

The atomistic details of binding between the *Ld*SMT and small molecules upon ligand binding were determined using the *BIOVIA* discovery studio visualizer v19.1.0.18287 (BIOVIA, San Diego, CA, USA) (Šudomová et al., 2019).

### 2.9 Quality Evaluation of Shortlisted Molecules

The inhibitory constant (*K_i_*) of the ligands was calculated from the binding energies of the selected compounds and the *Ld*SMT protein (Islam and Pillay, 2020). In addition, ligand efficiency (LE) metrics including ligand efficiency scale (LE_Scale), fit quality (FQ), ligand efficiency-dependent lipophilicity (LELP), and surface binding efficiency were also determined (Hopkins et al., 2014).

### 2.10 ADMET Properties and Drug-Likeness Assessment

The ADMET properties were determined using SwissADME (Daina et al., 2017) and the OSIRIS Property Explorer in Data Warrior (Sander et al., 2015). Pan-assay interference compounds (PAINS) (Daina et al., 2017) and synthetic accessibility (Daina et al., 2017) search using SwissADME (Daina et al., 2017) were performed to eliminate false positive compounds that possess good physiochemical properties as well as those with complex structures.

### 2.11 Prediction of Biological Activity of Selected Compounds

The biological activity of the selected compounds was predicted using prediction of activity spectra for substance (PASS) (Lagunin et al., 2000) with the simplified molecular input line entry system (SMILES) as inputs.

### 2.12 Molecular Dynamics Simulation

A 100-ns MD simulation was performed for the unbound *Ld*SMT and the protein–hit complexes using GROMACS 2018 (Van Der Spoel et al., 2005; Abraham et al., 2015). QtGrace (Dahiya et al., 2019) was used to plot the graphs generated from the MD simulation. The binding free energies of the complexes were calculated using MM-PBSA (Kumari et al., 2014). The energy contribution of each residue was also determined using g_MMPBSA. The graphs from the MM-PBSA computations were generated using the R programming language (Tippmann, 2014; Alkarkhi and Alqaraghuli, 2020).

### 2.13 Antileishmanial Exploration of Potential Leads

Structural similarity search of all the hits was done *via* DrugBank (Wishart et al., 2018) to identify drugs with potential antileishmanial activity and possible mechanisms of action from similar compounds.

## 3 Results and Discussion

### 3.1 Template Search

The 3D structure of *Ld*SMT is yet to be experimentally elucidated; therefore, the structure was modeled. A BLAST (Boratyn et al., 2013) search of the protein sequence of *Ld*SMT (SCMT1/GenBank ID: AAR92099.1) was performed *via* NCBI BLAST (Boratyn et al., 2013) to identify suitable identical templates to the *Ld*SMT. The search revealed 12 experimentally determined protein structures that are identical to the *Ld*SMT (**Supplementary Table 1**). The most widely used criteria in selecting a template is to choose the template with the highest sequence identity to the query sequence (Broni et al., 2021) and that was used for the modeling. However, the resolution at which the template protein structure was experimentally determined must also be taken into consideration. Also, the coverage of the template sequence to the query is another important factor. Herein, the *E*-value, sequence identity, query coverage, and the resolution of the 3D structures were used to select the most suitable templates as previously done (Meier and Söding, 2015; Haddad et al., 2020; Kwofie et al., 2021).

All the 12 identical protein structures had sequence identity less than 30% to the *Ld*SMT, and 5WP4 demonstrated the highest with an identity of 29.01% (**Supplementary Table 1**); however, 5WP4 had a relatively low coverage of 45% to the *Ld*SMT (**Supplementary Table 1**) and an *E*-value of 1 × 10^−11^. The 3BUS template had the least *E*-value of 6 × 10^−20^ and identity of 24.12%. The 3BUS protein has previously been used in modeling the SMT of *L. infantum* (Azam et al., 2014). Although 3BUS had a low resolution (2.65 Å), it was selected as one of the structures for modeling. 3BUS is the crystal structure of the rebeccamycin 4′-O-methyltransferase (RebM) in complex with S-adenosyl-l-homocysteine (Singh et al., 2008). On the other hand, the 4PNE template was also shortlisted as a suitable template due to its high coverage to the *Ld*SMT (61%), high resolution (1.50 Å), and sequence identity similar to that of 3BUS (24.15%). 4PNE is the SpnF enzyme in *Saccharopolyspora spinosa* involved in the biosynthesis of the insecticide spinosyn A (Fage et al., 2015; Jeon et al., 2017). SpnF has been reported to be structurally similar to S-adenosyl-L-methionine (SAM)-dependent methyltransferases (Fage et al., 2015).

Furthermore, a BLAST search *via* the SWISS-MODEL (Waterhouse et al., 2018) revealed that 4PNE covered residues 43 to 258 while 3BUS spanned from residues 47 to 276 of the *Ld*SMT. From residues 258 to 353 of the *Ld*SMT sequence, both templates do not share similarities with the *Ld*SMT. Thus, the protein structure with PDB ID 6UAK was selected in addition to 3BUS and 4PNE for the multitemplate homology modeling. The 6UAK shared similarity with the *Ld*SMT mostly from residues 100 to 345. 6UAK is a SAM-dependent methyltransferase (LahS_B_) from the *Lachnospiraceae* bacterium C6A11 (Huo et al., 2020). Both 3BUS and 6UAK templates, like the *Ld*SMT, are methyltransferases, while the 4PNE is a methyltransferase-like protein.

### 3.2 Structure Prediction of *Ld*SMT

An earlier study identified Modeller (Eswar et al., 2008) to predict the most accurate model of *L. infantum* sterol methyltransferase (Azam et al., 2014). Although the two organisms (*L. infantum* and *L. donovani*) belong to the same genus and SMT is highly conserved among *Leishmania* species (Goto et al., 2007), there was a need to model the structure of *Ld*SMT to ascertain its accuracy. Therefore, Modeller 10.2 (Eswar et al., 2008) was employed for modeling the structure of the *Ld*SMT.

Three modeling approaches were employed to predict the most reasonably accurate *Ld*SMT model. First, 3BUS was used as template to model five structures of the *Ld*SMT. Secondly, 4PNE was also used to model five different structures of the *Ld*SMT. Lastly, a multitemplate homology modeling approach was employed by using three templates comprising 3BUS, 4PNE, and 6UAK protein structures. For each approach, the best model was selected based on the DOPE score, which is an atomic distance-dependent statistical potential calculated from a sample of native protein structures (Shen and Sali, 2006). The DOPE scores were used to distinguish “good” models from “bad” ones with lower DOPE scores signifying a better model (Shen and Sali, 2006; Eswar et al., 2008).

#### 3.2.1 Structure Prediction Using 3BUS as Template

The 3BUS template with a sequence identity of 24.12% and coverage of 63% to the *Ld*SMT was used to generate five potential models of the *Ld*SMT (**Supplementary Table 1**). The five generated models (referred to as MOD3BUS1, MOD3BUS2, MOD3BUS3, MOD3BUS4, and MOD3BUS5) had genetic algorithm 341 (GA341) scores ranging from 0.87 to 0.99 (**Supplementary Table 2**). The GA341 score assesses the reliability of a model and has a determined threshold of 0.7. A model is said to be reliable when the GA341 score is higher than the cutoff (0.7) (Broni et al., 2021). For all the 3BUS-based models, the GA341 scores were greater than the cutoff signifying their reliability. Model MOD3BUS2 had the least DOPE score of −30,234.79297 and was selected as the most reasonable structure among the three models (**Supplementary Table 2** and **Supplementary Figure 2A**).

#### 3.2.2 Structure Prediction Using 4PNE as Template

**Figure 2:**
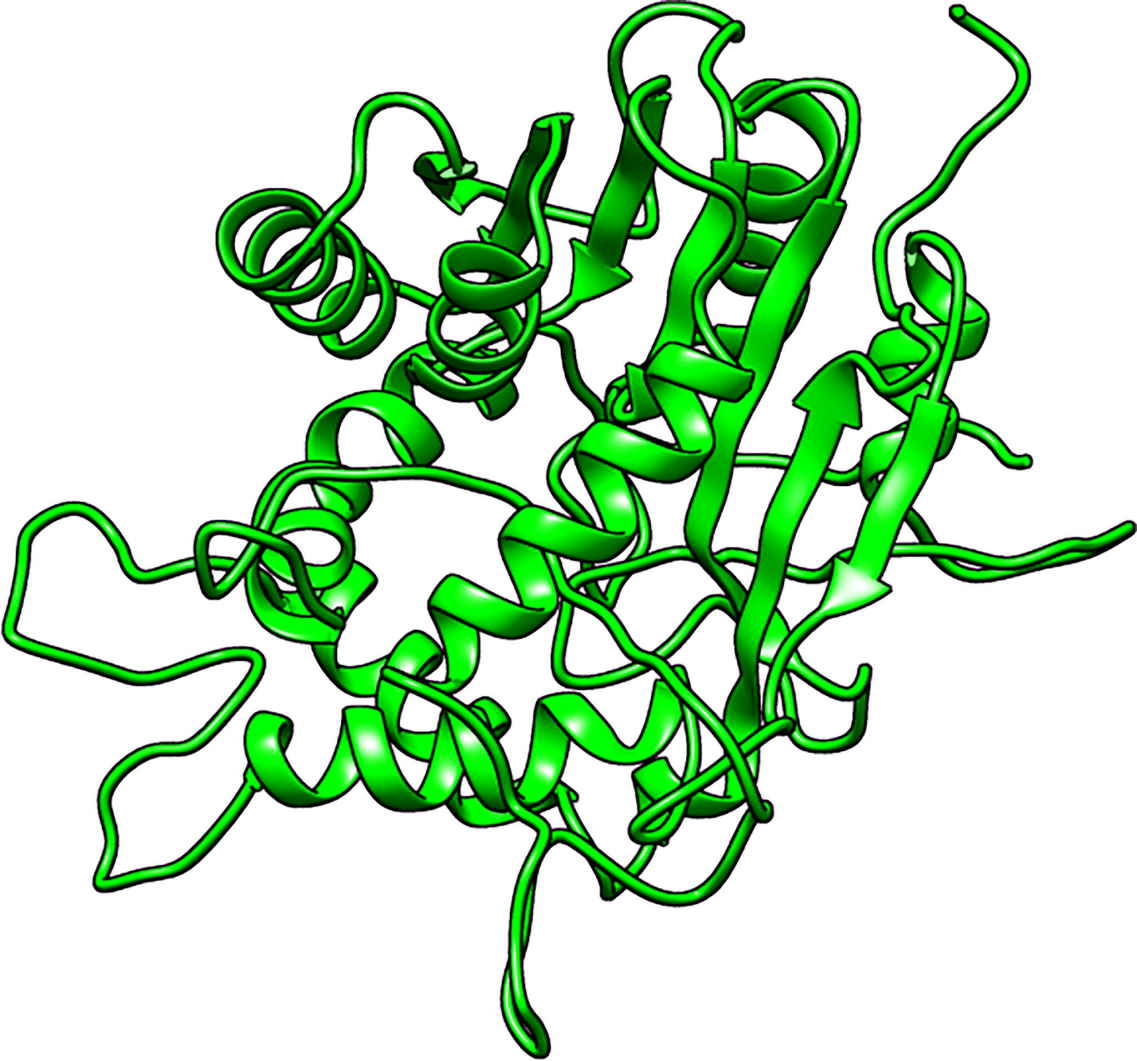
Cartoon representation of the structure of the selected *Leishmania donovani* 24-sterol methyltransferase (*Ld*SMT) model (MOD4PNE5).

A total of five structures were predicted using 4PNE as template. 4PNE had a sequence identity of 24.15% and a coverage of 61% to the *Ld*SMT (**Supplementary Table 1**). For the 4PNE-based models, the GA341 scores ranged between 0.73 and 0.98, signifying their high level of reliability (**Supplementary Table 2** and **Figure 2**). Model MOD4PNE5 had the least DOPE score of −31,608.05664 and was thus selected as the most reasonably accurate model for the 4PNE-based structures (**Supplementary Table 2** and **Figure 2**).

#### 3.2.3 Structure Prediction Using 3BUS, 4PNE, and 6UAK as Templates

For the third set of models, three templates comprising 3BUS, 4PNE, and 6UAK were used for the modeling because it has been reported that the use of multiple templates can help increase the accuracy of a model (Larsson et al., 2008). Model MOD3TEMP2 had a GA341 score of 0.64307, lower than the 0.7 cutoff. However, the other four models had good GA341 scores ranging from 0.7 to 0.94 (**Supplementary Table 2**). Among the multiple template-generated models, MOD3TEMP3 had the least DOPE score and was thus selected as the most accurate (**Supplementary Table 2** and **Supplementary Figure 2B**).

### 3.3 Validation of the Predicted Models

Next, validation and quality assessment of the predicted 3D structures were undertaken to obtain reasonable structures of the proteins. The best models from each of the three different approaches MOD3BUS2, MOD4PNE5, and MOD3TEMP3 were evaluated to select the most reasonably valid structure of the *Ld*SMT.

**Figure 3:**
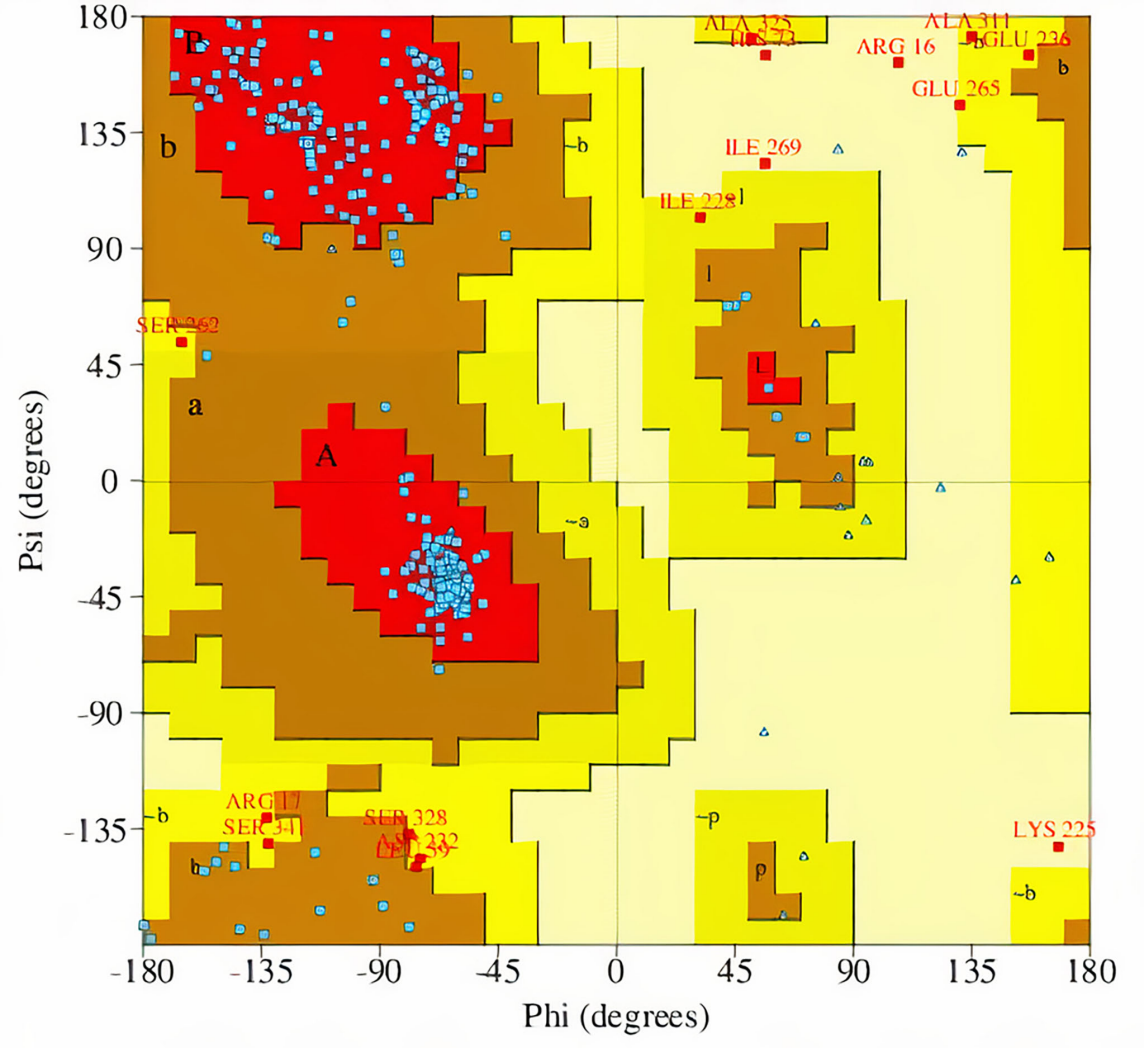
Ramachandran plot of the selected *Ld*SMT model (MOD4PNE5) obtained *via* PROCHECK. The percentages of residues in the most favored regions, additionally allowed regions, generously allowed regions, and disallowed regions are 84.9%, 10.3%, 3.5%, and 1.3%, respectively.

The percentage of residues in the most favored, additionally allowed, generously allowed, and disallowed regions in a Ramachandran plot determines the quality of protein structures. From the Ramachandran plots generated from PROCHECK, model MOD3BUS2 had 270 (86.8%), 32 (10.3%), 6 (1.9%), and 3 (1.0%) residues in the most favored, additionally allowed, generously allowed, and disallowed regions, respectively (**Table 1** and **Supplementary Figure 3A**). Model MOD3TEMP3 had 266 (85.5%), 31 (10.0%), 8 (2.6%), and 6 (1.9%) residues in the most favored, additionally allowed, generously allowed, and disallowed regions, respectively (**Table 1** and **Supplementary Figure 3B**). For the MOD4PNE5 model, 264 (84.9%) residues were in the most favored region, 32 (10.3%) in the additionally favored region, 11 (3.5%) in the generously allowed region, and 4 (1.3%) in the disallowed region (**Table 1** and **Figure 3**). The Ramachandran plot statistics of all three structures were comparatively close (**Table 1**) and are consistent with those of a previously modeled *Li*SMT structure using Modeller (Azam et al., 2014).

**Table 1:** Ramachandran plot statistics for the best models from the three modeling approaches.

Model	MOD3BUS2		MOD4PNE5		Refined MOD4PNE5		MOD3TEMP3	
	No. of residues	Percentage (%)	No. of residues	Percentage (%)	No. of residues	Percentage (%)	No. of residues	Percentage (%)
Most favored regions [A, B, L]	270	86.8	264	84.9	268	86.2	266	85.5
Additionally allowed regions [a, b, l, p]	32	10.3	32	10.3	34	10.9	31	10.0
Generously allowed regions [~a, ~b, ~l, ~p]	6	1.9	11	3.5	7	2.3	8	2.6
Disallowed regions	3	1.0	4	1.3	2	0.6	6	1.9
Non-glycine and non-proline residues	311	100.0	311	100.0	311	100.0	311	100.0

For all three models, the number of end residues (excluding Gly and Pro) = 2, glycine residues = 27, proline residues = 13, and the total number of residues = 353.

The qualities of the three structures were further assessed *via* SAVES v6.0 (Behera et al., 2021). The protein structure MOD3BUS2 had a VERIFY score of 62.61%, ERRAT quality factor of 41.5663, PROVE score of 11%, and four PROCHECK errors, one warning and three passes (**Supplementary Table 3**). For the MOD3TEMP3 model, ERRAT predicted an overall score of 45.858, VERIFY a score of 53.82%, PROVE a score of 9.6%, and five PROCHECK errors, one warning and two passes (**Supplementary Table 3**). Model MOD4PNE5 was predicted to have ERRAT, VERIFY, and PROVE scores of 46.9565, 62.04%, and 7.1%, respectively. MOD4PNE5 was also predicted to have four PROCHECK errors, two warnings and two passes (**Supplementary Table 3**) For a model to be considered high quality, 80% of its amino acids must have a score of 0.2 in the 3D-1D profile (VERIFY score). Although all the top 3 structures did not have a VERIFY score above 80%, MOD3BUS2 (VERIFY score of 62.61) and MOD4PNE5 (VERIFY score of 62.04) could be considered since a previous study has shown that a crystallized structure also performed poorly based on the VERIFY 3D quality indicator (Mora Lagares et al., 2020). For the ERRAT predictions, MOD4PNE5 had the best score and was also predicted using PROVE to be less erroneous (**Supplementary Table 3**). Generally, model MOD3TEMP3 exhibited the lowest quality scores from SAVES v6.0.

Based on the quality assessments, the protein structure MOD4PNE5 was selected as the most reasonable structure of the *Ld*SMT protein. Aligning the selected *Ld*SMT structure and the chain A of the 4PNE structure revealed a close similarity with a root mean square deviation (RMSD) of 0.356 Å (**Supplementary Figure 2C**). The quality of the selected model (MOD4PNE5) was further assessed *via* ProSA-web (Sippl, 1993; Wiederstein and Sippl, 2007). With a *Z*-score (Zhang and Skolnick, 1998) of −3.97, the *Ld*SMT structure was predicted to be of X-ray quality (**Supplementary Figure 4A**). The *Z*-score was used to indicate the overall protein quality (Zhang and Skolnick, 1998; Benkert et al., 2011). ProSA (Sippl, 1993; Wiederstein and Sippl, 2007) was used to plot the energies as a function of the amino acid sequence position for the *Ld*SMT protein (**Supplementary Figure 4B**). Positive energy values signify problematic or erroneous regions of the protein structure. The residues of the protein demonstrated relatively low energies until residue 230, where positive energy values were observed until the end of the sequence.

The selected *Ld*SMT structure was then refined using ModRefiner (Xu and Zhang, 2011). The refined structure of the selected *Ld*SMT model showed improved Ramachandran plot statistics with 268 (86.2%), 34 (10.9%), 7 (2.3%), and 2 (0.6%) residues in the most favored, additionally allowed, generously allowed, and disallowed regions, respectively (**Table 1**).

### 3.4 Active Site Prediction

CASTp was used to predict the plausible binding sites within the refined *Ld*SMT structure. CASTp predicted 65 potential binding sites of the *Ld*SMT protein. Ligand binding sites tend to involve the largest pockets or cavities on the protein (Laskowski et al., 1996; Liang et al., 1998); thus, pockets with relatively low areas and volumes, such that no ligand could fit, were not considered (Broni et al., 2021; Kwofie et al., 2021). A total of seven binding sites were shortlisted (**Supplementary Table 4**), which were visualized using PyMOL (PyMOL Molecular Graphics System, Version 1.5.0.4, Schrödinger, LLC) (Lighthall et al., 2010) and Chimera 1.16 (Pettersen et al., 2004). However, superimposing the *Ld*SMT on the 4PNE template revealed that pocket 1 was similar to the S-adenosyl-L-homocysteine (SAH) ligand’s binding site in the 4PNE protein (Fage et al., 2015). Surprisingly, pocket 1 for *Ld*SMT was predicted to have no opening in Chimera 1.16 (Pettersen et al., 2004), leaving pockets 2 to 7 as the most plausible binding cavities of the *Ld*SMT (**Supplementary Table 4**). Pockets 5 and 7 also overlapped and occupied the same region (**Supplementary Table 4**).

### 3.5 Scaffold Hopping *via* BRUSELAS and Molecular Docking

Following the protocols of BRUSELAS, 100 ligands were generated using the ChEMBL database (Davies et al., 2015) with varying degrees of similarities to 22,26-azsterol. Out of the 100 ligands, 17 were identified to possess unique scaffolds devoid of the steroidal nucleus present in the 22,26-azasterol. The total score (comprising a combination of WEGA, LiSiCA, Screen3D, and OptiPharm) ranged from 0.38458 to 0.65814. Ten of the molecules with different scaffolds and varying scores are presented (**Supplementary Table 5**).

A search *via* the PubChem (Kim et al., 2021), ZINC15 (Sterling and Irwin, 2015), DrugBank (Wishart et al., 2018), and ChemSpider databases (Pence and Williams, 2010) generated 1,342 derivatives of all 17 scaffolds. The 1,370 compounds consisting of 17 scaffolds, 1,342 derivatives, 22,26-azasterol, 7 other known inhibitors (labeled X1–X7), and 3 already known drugs (amphotericin B, miltefosine, and paromomycin) were screened against an energy minimized *Ld*SMT using a grid box of (91.445 × 73.502 × 78.352) Å^3^ with the center at (72.200, 58.009, 13.302) Å to cover the protein. Screening 1,370 compounds against the active site of the protein identified 25 hits which were selected based on the binding affinities and orientation within the binding site of the protein.

Among the three drugs used in leishmaniasis treatment, amphotericin B had the least binding energy of −5.3 kcal/mol followed by paromomycin (−5.0 kcal/mol) and miltefosine (−4.0 kcal/mol). Interestingly, all the known inhibitors had binding energies lower than the three drugs signifying a higher binding affinity to *Ld*SMT. The binding energies of −5.9, −6.2, and −6.5 kcal/mol were obtained for X6, X3, and X4, respectively. The least binding energy of −7.7 kcal/mol was observed for X5 comparable to 22,26-azasterol (−7.6 kcal/mol). *In-vitro* studies reported that 22,26-azasterol inhibited *L. donovani* intracellular amastigotes and *Trypanosoma brucei* subsp. *brucei* with IC_50_ values of 8.9 and 1.76 μM, respectively (Magaraci et al., 2003; Gros et al., 2006), supporting the results reported herewith. Compounds X1, X7, and X2 had binding energies of −7.3, −7.2, and −7.0 kcal/mol, respectively (**Supplementary Table 6**). Similarly, *in-vitro* studies revealed that X1, X2, X3, X4, X5, X6, and X7 inhibited the growth of *Leishmania* parasites with IC_50_ less than 10 μM, except for X6 and X7 which were found to suppress growth with IC_50_ values of 28.6 and 30 μM, respectively (Magaraci et al., 2003; Lorente et al., 2004; Andrade-Neto et al., 2016; Torres-Santos et al., 2016).

The 12 best hits out of the 25 selected from the scaffold hopping had lower binding energies compared with the three drugs (**Supplementary Table 6**). In addition, the binding energies of these ligands were also found to be comparable to the known inhibitors with S1 showing the least binding energy of −9.0 kcal/mol. The closest to this ligand were S2, S3, and S4 with binding energies of −8.9, −8.8, and −8.7 kcal/mol, respectively (**Supplementary Table 6**). Compounds S12, S11, and S10 had the highest binding energies among the 12 best compounds with binding energies of −7.0, −7.0, and −7.2 kcal/mol, respectively. In addition, the binding energies of compounds S9 (−7.3 kcal/mol), S8 (−7.4 kcal/mol), and S7 (−7.4 kcal/mol) were also obtained. Comparable binding energies to the two lowest binding energies of the known inhibitors were obtained for S5 (−7.7 kcal/mol) and S6 (−7.6 kcal/mol).

### 3.6 *De-Novo* Design *via* e-LEA3D and Molecular Docking

The *de-novo* drug design is the generation of novel chemical entities that fit a set of constraints using computational algorithms (Schneider and Schneider, 2016). Despite the challenges of synthetic accessibility associated with this method, the application of *de-novo* drug design leads to the development of drug candidates in a cost- and time-efficient manner (Mouchlis et al., 2021). In addition, the *de-novo* design generates novel compounds with improved biological activity (Mouchlis et al., 2021). A number of studies have been undertaken for the *de-novo* design of inhibitors against plausible targets (Kranthi et al., 2018; Islam and Pillay, 2020; Pathania et al., 2021). A previous study selected a lead molecule based on the least binding energy as well the accurate pose of the ligand within the protein binding pocket for the *de-novo* design of inhibitors (Pathania et al., 2021). A similar approach was used in the identification of promising anti-DNA gyrase antibacterial compounds (Islam and Pillay, 2020). Ligands with very low binding energies and accurate pose have the potential to inhibit the receptor.

Among the compounds obtained from scaffold hopping, the well-known heterocyclic quinolinone and the phenylpiperazine/phenylpiperidine moieties found in several bioactive compounds were present. These compounds with diverse pharmacological potencies have been explored for various ailments including leishmaniasis (Kshirsagar, 2015; Chanquia et al., 2019; Mishra et al., 2021). One of such derivatives is 2-(4-(4,6-di(piperidin-1-yl)-1,3,5-triazin-2-ylamino)phenyl)-2-methyl2,3-dihydroquinazolin-4(1H)-one which had an IC_50_ value of 0.65 μM against intracellular amastigotes when compared with miltefosine (IC_50_ of 8.4 μM) (Sharma et al., 2013), corroborating the fact that *de-novo* drug design could result in novel scaffolds as potential antileishmanial agents.

**Figure 4:**
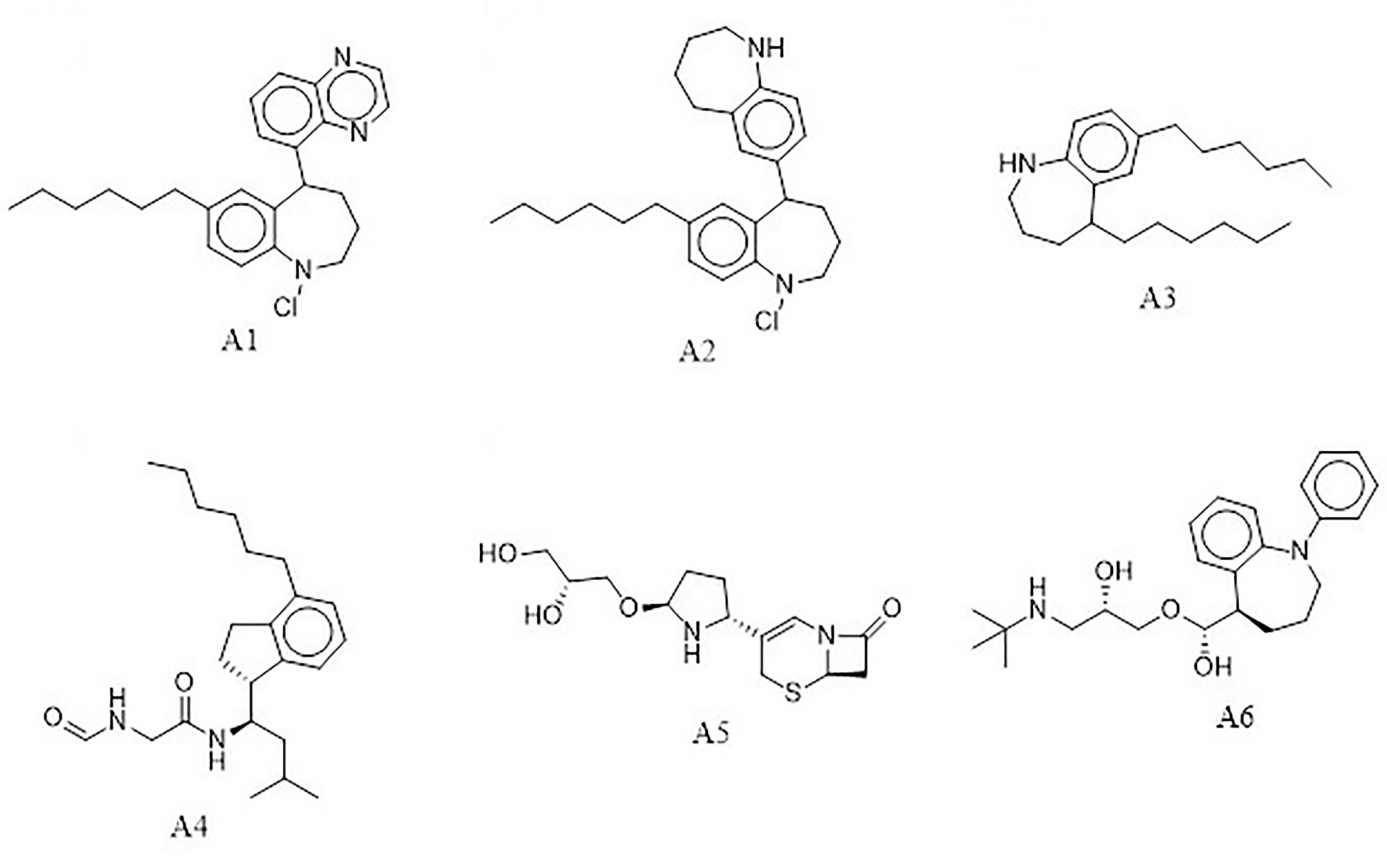
Top hits from *de-novo* drug design using the e-LEA3D.

The e-LEA3D was used to generate 155 potential novel compounds against *Ld*SMT after using protein–S1, S2, S3, S4, and 22,26-azasterol complexes. They were filtered based on Lipinski’s rule of five (Benet et al., 2016) and redundancy to generate 78 ligands that were subjected to molecular docking studies. The six best hits (**A1**, **A2**, **A3**, **A4**, **A5**, and **A6**) (**Figure 4**) were selected for downstream analysis. The search *via* public databases including PubChem (Kim et al., 2021) and ChemSpider (Pence and Williams, 2010) showed that the six ligands do not have duplicates.

The docking analysis of the six compounds revealed the binding energies of **A1** (−8.4 kcal/mol), **A2** (−7.5 kcal/mol), and **A3** (−7.2 kcal/mol), while **A4**, **A5**, and **A6** had −7.0 kcal/mol. Though **A1**, **A2**, **A3**, and **A6** have similar core structures, they had different binding energies (**Table 2**). Interestingly, using AutoDock Vina, compounds that had shown binding energies ≤−7.0 kcal/mol have been found to demonstrate significant inhibitory activities against the parasite of consideration (Chang et al., 2007; Wyllie et al., 2018; Tabrez et al., 2021). In lieu of this, the predicted compounds may have the potential of suppressing *Ld*SMT since the binding energies were lower than −7.0 kcal/mol. Altogether, compounds **A1**, **A2**, **A3**, **A4**, **A5**, and **A6** showed binding energies lower than amphotericin B, miltefosine, and paromomycin. Similarly, all the compounds had binding energies comparable to the known inhibitors and, therefore, have the potential of attenuating *Ld*SMT.

**Table 2:** Binding energies and predicted interacting residues in the *Ld*SMT–hit complexes.

Compounds	Binding energies (kcal/mol)	Interacting residues	
		Hydrogen bonds	Hydrophobic bonds
22,26-Azasterol	−7.6	Glu102, Gly200	Phe100, Lys198, Pro199
**A1**	−8.4	Asp58	Arg89, Tyr92, Ala95, Ala96, Leu123
**A2**	−7.5	–	Phe84, Glu85, Ala88, Arg89, Tyr92
**A3**	−7.2	–	Ala88, Arg89, Tyr92, Phe93, Ala96, Phe264
**A4**	−7.0	Cys202	Gly98, Phe100, Asp104, Tyr343, Ile344
**A5**	−7.0	Arg222, Lys317	Val26, Ala30, Phe33, Phe37, Met52, Ile224
**A6**	−7.0	Asp58	Phe33, Ile258, Phe264

### 3.7 Characterization of Binding Interactions

Compounds with similar activity against a receptor may possess identical chemical features in sterically consistent locations within the pocket of a macromolecule (Held et al., 2011; Du et al., 2016). Most of the compounds including 22,26-azasterol and its derivatives were observed to interact with residues Asp58, Ala88, Arg89, Tyr92, Glu85, Phe93, Phe100, Glu102, Lys198, Pro199, and Gly200, which lined binding pockets 5 and 7 (**Supplementary Tables 4** and **6**). A similar study involving *L. infantum* SMT, however, showed the ligands to interact with residues Tyr1, Gly4, Gln5, Gly45, Gly47, Asn67, Asn68, Gln72, and Ile112 within the binding pocket of the receptor (Azam et al., 2014). The nature of interactions included *pi*–anion, *pi*–*pi* stacking, *pi*–alkyl, *pi*–sigma, carbon–hydrogen, and hydrogen bonds similar to the other study (Azam et al., 2014). Among all the ligands, only amphotericin B had five hydrogen bonds, with residues Asp31, Phe307, Val308, Arg309, and Leu310 found to line pocket 2. Compounds X1 and X5 formed two hydrogen bonds each with *Ld*SMT. Compound X1 interacted with pockets 5 and 7 and residues Asp172 and Gly200, while X5 interacted with pocket 4 and residues Asn12 and Thr319 *via* hydrogen bonds (**Supplementary Table 6**).

In addition, compounds X2, X6, X7, S1, S5, S9, S10, and S12 docked into the binding pocket of the receptor but showed no hydrogen bond interactions with any of the residues. The compound 22,26-azasterol formed two hydrogen bonds with Glu102 and Gly200 as well as hydrophobic interactions with Phe100, Lys198, and Pro199. Moreover, while paromomycin, S2, S3, S4, S5, and S11 formed a hydrogen bond with Arg89, that of S6 and S7 showed a similar interaction with Asp58. Apart from paromomycin, all the other ligands which interacted with Arg89 had low binding energies, implying that it might be critical for binding. The known drugs and inhibitors formed hydrophobic interactions with one or more of the residues Phe100, Met101, Asp104, Asp172, Pro199, Gly200, Thr201, Tyr343, and Ile344, while the selected S-class compounds showed hydrophobic interactions with one or more of the residues Asp58, Ala88, Arg89, Tyr92, Phe93, and Phe264 (**Supplementary Table 6**).

**Figure 5:**
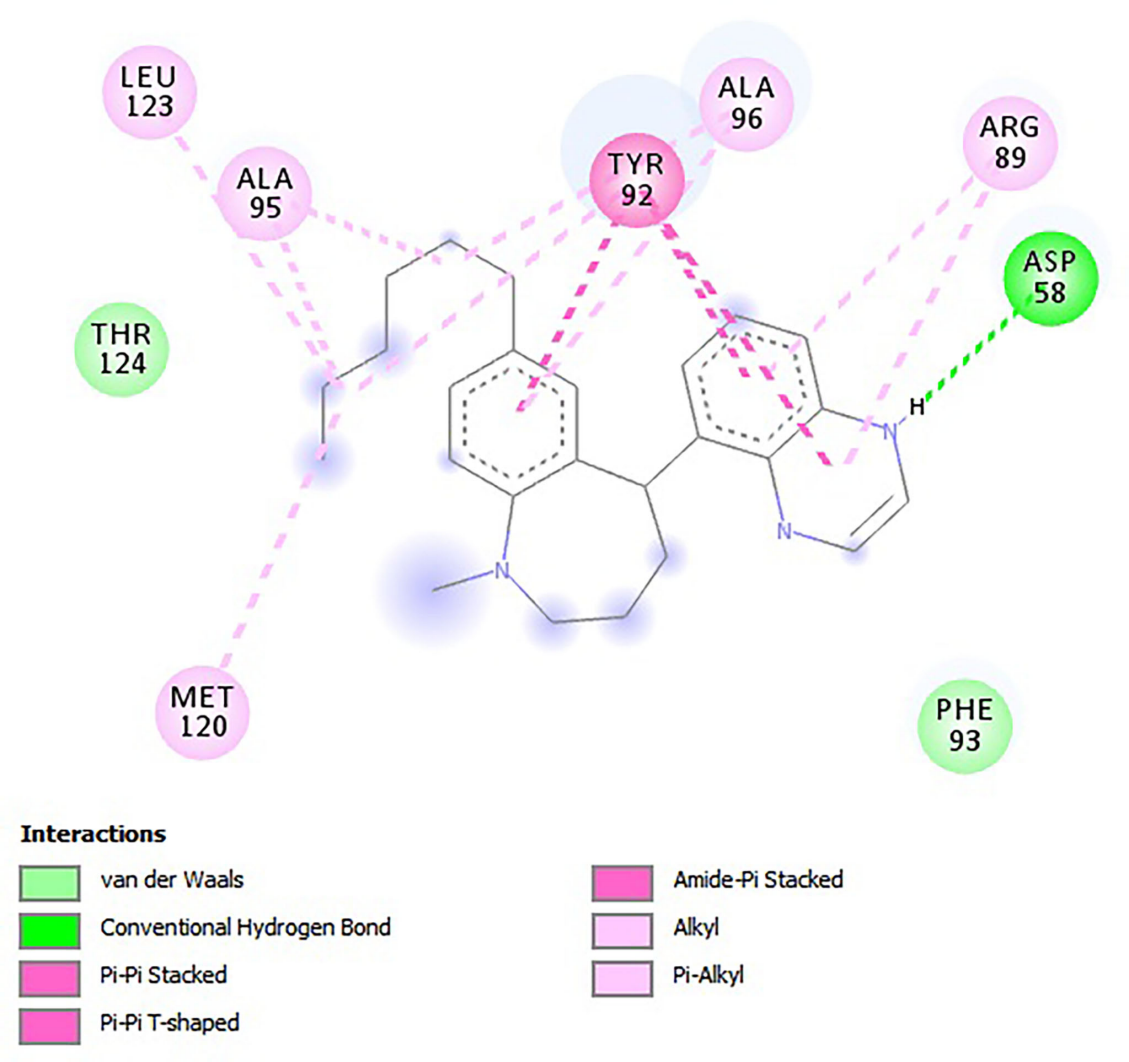
2D interaction profile of the *Ld*SMT–A1 complex as visualized in Discovery Studio.

For the e-LEA3D-generated hits, apart from **A2** and **A3** which were predicted not to form hydrogen bond interactions with any amino acid residues, the remaining four exhibited hydrogen bonding with at least one of the amino acid residues of *Ld*SMT (**Table 2**). This notwithstanding, all six formed hydrophobic interactions with the *Ld*SMT protein. The hydrophobic interactions for **A1** were with residues Arg89, Tyr92, Ala95, Ala96, and Leu123 (**Figure 5** and **Table 2**), and those for **A2** were with Phe84, Glu85, Ala88, Arg89, and Tyr92 (**Table 2** and **Supplementary Figure 5A**). The ligand **A3**, on the other hand, formed hydrophobic interactions with Ala88, Arg89, Tyr92, Phe93, Ala96, and Phe264 (**Table 2** and **Supplementary Figure 5B**). Furthermore, both **A4** (**Supplementary Figure 5C**) and **A6** (**Supplementary Figure 5E**) formed hydrogen bonding with Cys202 and Asp58, respectively, while **A5** (**Supplementary Figure 5D**) formed two hydrogen bonding interactions with Arg222 and Lys317.

### 3.8 Physicochemical, Pharmacological, and Toxicity Profiling

A druggable candidate must be able to reach the site of action in the body at optimized concentrations (Hughes et al., 2011). Predictions of pharmacological and physicochemical parameters are essential because they offer clues as to whether the molecule could reach the active site in the desired concentration and remain there to elicit the required biological response. The physicochemical and pharmacokinetic parameters of the six compounds were assessed using SwissADME (Hughes et al., 2011). Physicochemical profiling assessed both Lipinski’s rule of five (RO5) and Veber’s rule to determine if the chemical compounds with certain pharmacological or biological activity have chemical and physical properties to make them orally active (Lipinski et al., 2001; Veber et al., 2002). **A5** and **A6** were found to obey both Lipinski’s and Veber’s rules, and **A1**, **A2**, **A3**, and **A4** violated one of the two rules (**Supplementary Table 7**).

Solubility, an important physicochemical property, was predicted to assess the bioavailability and bioactivity of the hit compounds (van den Anker et al., 2018). Many lead compounds have failed to reach clinical trials despite being potent because of low bioactivity attributed to insufficient solubility, making solubility predictions critical in the early stages of drug design (Das et al., 2022). Compounds **A4**, **A5**, and **A6** were, however, predicted to be soluble compared with **A1**, **A2**, and **A3** whose solubility can be improved upon by structural modification (Das et al., 2022).

Next, molar refractivity (MR) was assessed to give valuable information on the pharmacokinetics and pharmacodynamics of the compounds. This is governed by different interactions in solution such as drug–solvent, drug–drug, and drug–co-solute interactions (Sawale et al., 2016). The compounds passed for molar refractivity as the predicted values for MR (**Supplementary Table 7**) were all within the acceptable range of 40 to 130. Since compounds with topological polar surface area (tPSA) not more than 140 Å^2^ are considered to have good oral bioavailability, all compounds are predicted to be orally active and have good bioavailability.

Furthermore, synthetic accessibility was explored to evaluate the synthetic feasibility of the *de-novo* hits and has gained importance in the prioritization of compounds in drug design (Ertl and Schuffenhauer, 2009). Many druggable candidates especially *de-novo* constructed chemical entities are unable to reach clinical trials due to their molecular complexity coupled with difficulty in synthesis (Huang et al., 2010; de Souza Neto et al., 2020). SwissADME (Daina et al., 2017) predicted all the six compounds to possess synthetic accessibility less than 6 implying easy synthesis.

The PAINS of the chemical compounds was investigated to establish whether they will react non-specifically with numerous biological targets (Baell, 2016). A number of PAINS compounds include toxoflavin, isothiazolones, hydroxyphenyl hydrazones, curcumin, phenol-sulfonamides, rhodanines, enones, quinones, and catechols (Baell and Walters, 2014). It is reported that about 5% of US FDA-approved drugs obtained from natural and synthetic drugs still contain PAINS-recognized substructures (Baell and Nissink, 2017). All the compounds were predicted not to contain PAINS substructures.

Pharmacokinetics studies were used to evaluate the time course of absorption, distribution, metabolism, and excretion of the selected hits (Luer and Penzak, 2016). The parameters measured were blood–brain barrier (BBB), gastrointestinal absorption (GI), and permeability glycoprotein (P-gp). Compounds predicted to permeate the BBB have the potential to bind to relevant receptors of the brain to activate signal pathways (Banks, 2009). Four of the compounds (**A1**, **A2**, **A3**, and **A5**) were predicted not to cross the blood–brain barrier, while **A4** and **A6** were predicted to cross the BBB to attach to the receptors in the brain to elicit a biological response. GI absorption was probed to investigate whether the hit compounds will be absorbed into the bloodstream after metabolism when orally administered (Löbenberg et al., 2013). All ligands were predicted to have a high GI absorption score except **A2** and **A3** (**Supplementary Table 7**). Another pharmacokinetic parameter considered for this study was to explore whether the hits generated were P-gp substrates as compounds predicted to inhibit P-gp result in their increased bioavailability (Lin and Yamazaki, 2003; Prachayasittikul and Prachayasittikul, 2016). The six compounds were screened for their P-glycoprotein binding affinity and they were all predicted to be substrates except **A4**.

The toxicity profiles of all six compounds were predicted using OSIRIS Property Explorer in Data Warrior (Sander et al., 2015). Toxicity prediction has become very critical in the development of drugs as over 45% of drug candidates fail due to toxicity deficiencies (Van Norman, 2019). Moreover, between 1953 and 2013, as many as 462 medicinal products were withdrawn from the market due to adverse drug reactions (Onakpoya et al., 2016). Toxicity profiling considered for this study was mutagenicity, carcinogenicity, irritancy, and reproductive effects. Of the six compounds, only **A1** was predicted to be tumorigenic. The rest neither were mutagenic nor possessed any irritant or reproductive effects (**Supplementary Table 8**). Among all the compounds under consideration, only X6 was predicted to possess reproductive effects. Overall, the predictions indicate that all molecules may have safe pharmacokinetic and pharmacodynamic profiles except for **A1**, which would require structural modification to improve its pharmacological properties. For instance, the prediction showed that replacement of the chlorine substituent with a hydrogen atom could render the **A1** analog non-tumorigenic.

### 3.9 Bioactivity Prediction

The open Bayesian machine learning technique, PASS, was used to predict the biological activity of the ligands based on the structure–activity relationship between the selected hits and the training set of compounds of known biological activity (Lagunin et al., 2000; Parasuraman, 2011). A ligand is said to be biologically active and requires experimental validation if the probability of activity (Pa) is greater than the probability of inactivity (Pi) (Basanagouda et al., 2011). Among the six compounds, **A3** was predicted to possess antileishmanial properties with a Pa of 0.362 and a Pi of 0.066 and also dermatological properties with a Pa of 0.32 and a Pi of 0.091. Compounds **A5** and **A6** were also predicted as dermatologic, with Pa values of 0.205 and 0.249 and Pi values of 0.162 and 0.120, respectively. The results may suggest that **A3**, **A5**, and **A6** might be beneficial in treating post-kala-azar leishmaniasis (Momeni et al., 2003; Ali et al., 2012).

Compounds **A1** (Pa of 0.571 and Pi of 0.111), **A3** (Pa of 0.774 and Pi of 0.026), and **A6** (Pa of 0.615 and Pi of 0.079) were also predicted as mucomembrane protectors. A recent *in-vitro* study revealed that butein acting as a mucomembrane protector on human cells increased immunity against pathogenic infections (Satari et al., 2021). This may suggest that the compounds have the potential of boosting the immune system to prevent disease exacerbation. Compound **A1** was predicted as an indolepyruvate C-methyltransferase inhibitor with a Pa of 0.226 and a Pi of 0.074. Compound **A3** was also predicted to be phenol O-methyltransferase, histamine N-methyltransferase, and acetylserotonin O-methyltransferase inhibitors with Pa values greater than Pi.

### 3.10 Quality Assessment

The inhibitory constant (*K_i_*) and other parameters such as LE, LE_Scale, FQ, binding efficiency index (BEI), surface efficiency index (SEI), and LELP were calculated (**Supplementary Table 9**).

*K_i_* is the concentration required to produce half-maximum inhibition and, hence, an indicator of the potency of a ligand (Fisar et al., 2010). Computation of *K_i_* for the protein–ligand complexes was obtained using Equation (1), where *R* is the molar gas constant (1.987 × 10^−3^ kcal/K mol^−1^) and *T* (298.15 K) is the absolute temperature (Du et al., 2016).


(1) $$K_{i}=e^{\frac{-\Delta G}{RT}}$$

The *K_i_* predicted for the ligands was low (**Supplementary Table 9**), hence has the capacity to be lead-like with possible high potency (Reynolds and Reynolds, 2017).

LE is a value that expresses the binding energy of a compound normalized by the compound’s size and expressed by the number of heavy (non-hydrogen) atoms (Hopkins et al., 2004). This property is important to consider in screening for hits as larger compounds tend to show greater binding energy due to a large number of interactions but may not necessarily be the most efficient binder (Hevener et al., 2018). The LE was computed using Equation (2), where BE is the binding energy and NHA is the number of heavy atoms (Abad-Zapatero et al., 2010).


(2) $$\text{LE}=\frac{-\text{BE}}{\text{NHA}}$$

Interestingly, all the ligands except **A1**, **A3**, and **A5** were within the optimal range (LE < 0.3 kcal/mol/HA) (Schultes et al., 2010) for the ligand efficiency of lead-like molecules.

Results from the computation of LE being size-dependent may not be a true reflection of the binding energy of the compound, and therefore, ligand efficiency scaling (LE_Scale), a size-independent parameter that compares ligands with the help of an exponential function to the maximal LE values, is required (Reynolds et al., 2007). LE_Scaling was computed using Equation (3), where NHA is the number of heavy atoms (Reynolds et al., 2007).


(3) $$\text{LE}\_\text{Scaling}=0.873{\text{e}}^{-0.026\times \text{NHA}}-0.064$$

Potential lead-like molecules are suggested to have an LE_Scale lower than 0.3 (Islam and Pillay, 2020). The LE_Scale values of all six molecules were, however, predicted to be in the range of 0.3 to 0.5 with **A5** having the highest LE_Scale of 0.455.

FQ is another size-independent parameter that determines the optimal ligand binding within the receptor active site (Schultes et al., 2010), and is computed using Equation (4) (Schultes et al., 2010).


(4) $$\text{FQ}=\frac{\text{LE}}{\text{LE}\_\text{Scale}}$$

FQ scores range from 0 to 1 with values close to 1 signifying an optimal ligand binding (Schultes et al., 2010). All the compounds were predicted to have an FQ score above 0.7 except **A6** (0.695), implying a stronger ligand binding. With the predicted FQ being close to 1, it suggests an optimal ligand binding.

The LELP, on the other hand, assesses the binding energy of a compound in relation to the compound’s lipophilicity (Hevener et al., 2018). LELP is a parameter used in drug design and development to evaluate the quality of compounds by linking potency and lipophilicity in an attempt to estimate drug-likeness (Edwards and Price, 2010). Equation (5), where log*P* is the lipophilicity, was used in calculating the LELP of the compounds (Schultes et al., 2010).


(5) $$\text{LELP}=\frac{\text{logP}}{\text{LE}}$$

The recommended range for promising molecules for LELP was >3 (Schultes et al., 2010). All the analogs had LELP above 4 (**Supplementary Table 9**) suggesting an optimized affinity with respect to lipophilicity.

BEI and SEI are two alternative metrics that are also used to compare the activity of molecules according to size and area (Schultes et al., 2010). Binding efficiency index is defined by BEI = p(IC_50_)/MW, where MW is the molecular weight (Schultes et al., 2010). The relation in Equation (6) was used in computing the BEI of the ligands (Abad-Zapatero et al., 2010).


(6) $$\text{BEI}=\frac{-{\text{logK}}_{\text{i}}}{\text{MW}\left(\text{kDa}\right)}$$

SEI, on the other hand, is defined by SEI = p(IC_50_)/PSA, where PSA is the polar surface area of the ligand (Abad-Zapatero et al., 2010). Calculation of SEI was done using Equation (7) (Abad-Zapatero et al., 2010).


(7) $$\text{SEI}=\frac{-{\text{logK}}_{\text{i}}}{\left(\text{PSA}/100\right)}$$

By rule of thumb, potential inhibitors must approximately have the same BEI and SEI values (Abad-Zapatero, 2007). Compounds **A1**, **A4**, and **A6** were predicted to have BEI equal to SEI. Altogether, the parameters predicted for all the compounds were mostly within the acceptable range prompting the need for experimental analysis.

### 3.11 Molecular Dynamics Analysis

MD simulation is a computer simulation method for analyzing the physical movements of atoms and molecules (Hollingsworth and Dror, 2018). Of great concern for the MD simulation is how a biomolecular system responds to some perturbation within a short period of time (Karplus and Kuriyan, 2005; Hollingsworth and Dror, 2018). Due to its usefulness, a number of drug design studies have explored molecular dynamics simulation to analyze and validate the binding poses, stability of the complexes, and binding affinity of selected hits within the binding pocket of the receptor (Agyapong et al., 2021; Gupta et al., 2021; Pathania et al., 2021). To check the relative stability of each complex using a 100-ns time span MD simulation, parameters such as RMSD, root mean square fluctuation (RMSF), and radius of gyration (Rg) were computed.

#### 3.11.1 The Root Mean Square Deviation of the Unbound *Ld*SMT and the Complexes

**Figure 6:**
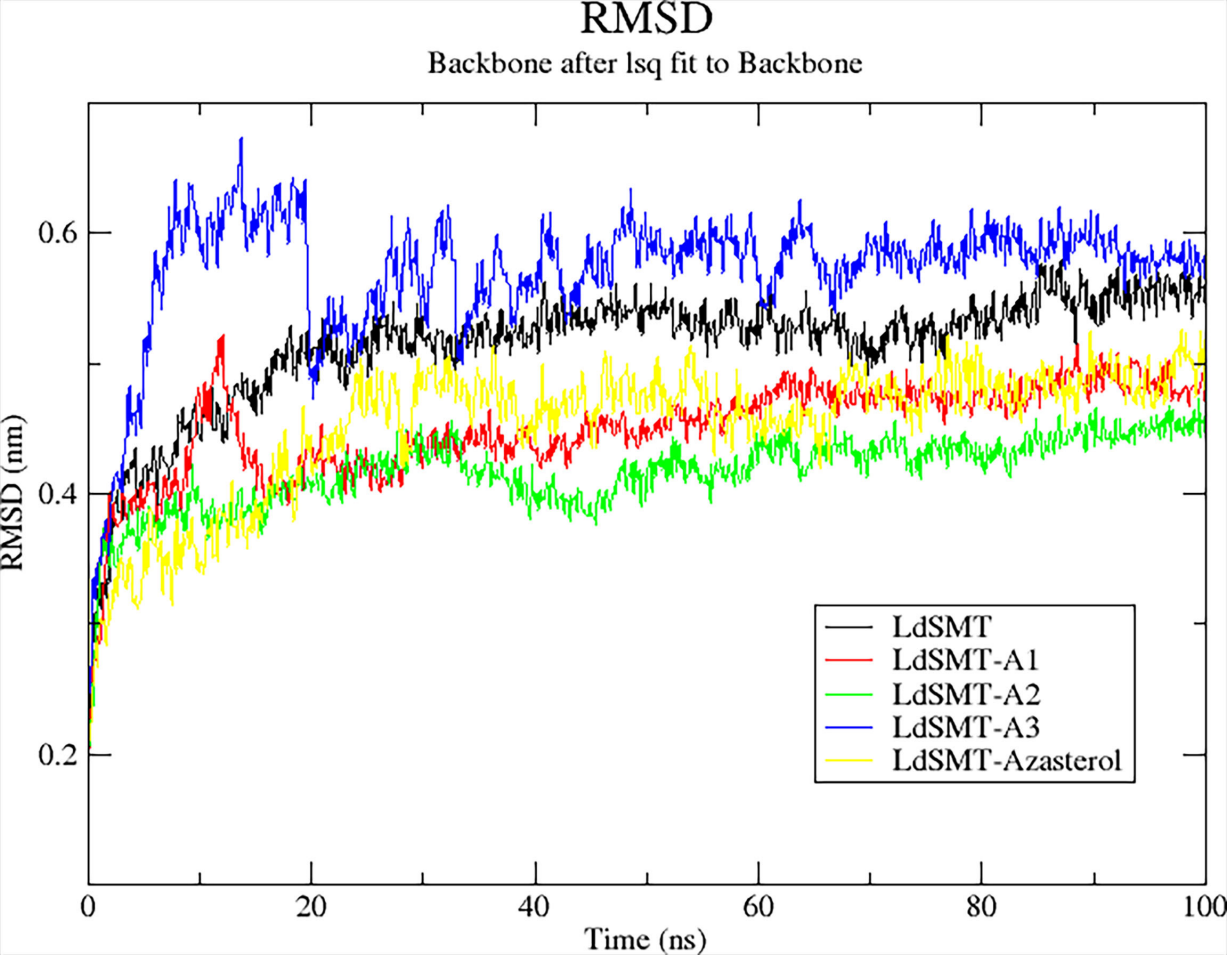
Root mean square deviation (RMSD) plot of 100 ns molecular dynamics (MD) simulations of the *Ld*SMT–ligand complexes using GROMACS.

The RMSD trajectory was used to evaluate the stability of the protein–ligand complexes, and the plot shows the system was equilibrated in the range of 0 to 0.7 nm. Averagely, the unbound *Ld*SMT was observed to rise steadily from 0.28 nm until about 0.42 nm for 20 ns before stabilizing thereafter (**Figure 6**). Comparatively, there is no significant fluctuation observed in any of the protein–ligand complexes except for the *Ld*SMT*–***A3** complex, which rose from 0.28 to 0.62 nm during 10 ns and then stabilized for the next 10 ns and dropped to about 0.5 nm. It then rose slightly to 0.56 nm and then stabilized after 40 ns. Across the board, the *Ld*SMT–**A2** complex had high rigidity and the frames of each complex depict a tight structural packing across the whole protein influencing the low RSMD values of *Ld*SMT.

#### 3.11.2 Radius of Gyration of the *Ld*SMT and the Complexes

The radius of the gyration plot, a graph of Rg against simulation time, is used to analyze the compactness and folding of the unbound protein and complexes during the molecular dynamics simulations. The Rg graph obtained showed that all the complexes had low Rg values implying that the ligands formed a stable and compact complex (Liao et al., 2014; Pandey et al., 2020; Sinha and Wang, 2020). All the complexes except for **A3** had a steady decline in Rg to about 40 ns before stabilizing afterward. Rg values for both unbound protein and the complexes were between 1.925 and 2.75 nm (**Supplementary Figure 6A**). The Rg trajectory of each complex demonstrated that each ligand forms a stable bond with the *Ld*SMT.

#### 3.11.3 The Root Mean Square Fluctuation of the *Ld*SMT and the Complexes

The RMSF was explored to investigate which amino acids within the binding site of the receptor interacted with the ligand resulting in the stability of the protein–ligand complex (Farmer et al., 2017). The RMSF plots showed that all the hit compounds caused fluctuations in similar positions of the protein target. The plots revealed that the amino acid residues between 15 and 100, 200 and 250, and 280 and 320 (**Supplementary Figure 6B**) fluctuated for all complexes and are predicted to be involved in the stability of the complexes (Dong et al., 2018). However, the highest fluctuation was observed around regions 200–280 for **A2** and **A3** implying it could be involved in ligand binding.

### 3.12 MM-PBSA Free Energy Computations

#### 3.12.1 Binding Energy Assessment Scores

The free energy of binding of all the protein–ligand complexes was calculated using the MM-PBSA continuum solvation method (Kumari et al., 2014). The MM-PBSA was employed to find the free energies of the bound complexes. Ligand **A1**, which was predicted to have the least binding energy from AutoDock Vina, was shown to have the lowest free binding energy of −282.550 kJ/mol (**Table 3**) to the *Ld*SMT. Among the three top compounds, only **A3** exhibited free binding energy greater than that of the reference candidate, 22,26-azasterol (**Table 3**). The dominating interaction per contribution to the free energy was electrostatic forces of attraction ranging from −333 to −11 kJ/mol followed by the van der Waals interactions.

**Table 3:** MM-PBSA energy assessment of the *de-novo* hits and 22,26-azasterol.

Complex	Δ*G*_vdW_ (kJ/mol)	Δ*G*_ele_ (kJ/mol)	Δ*G*_ele, sol_ (kJ/mol)	Δ*G*_SASA_ (kJ/mol)	Δ*G*_bind_ (kJ/mol)
**A1**	−254 ± 20.790	−276.921 ± 49.836	268.533 ± 68.275	−19.358 ± 1.489	−282.550 ± 35.346
**A2**	−165 ± 44.344	−333.723 ± 82.848	371.954 ± 91.519	−14.820 ± 3.992	−142.568 ± 47.076
**A3**	−49.793 ± 41.867	−11.805 ± 11.107	25.031 ± 43.156	−4.827 ± 4.582	−41.394 ± 44.095
22,26-Azasterol	−0.047 ± 0.042	−56.829 ± 37.192	−15.475 ± 37.519	0.045 ± 2.716	−72.305 ± 59.057

#### 3.12.2 Per-Residue Energy Decomposition

**Figure 7:**
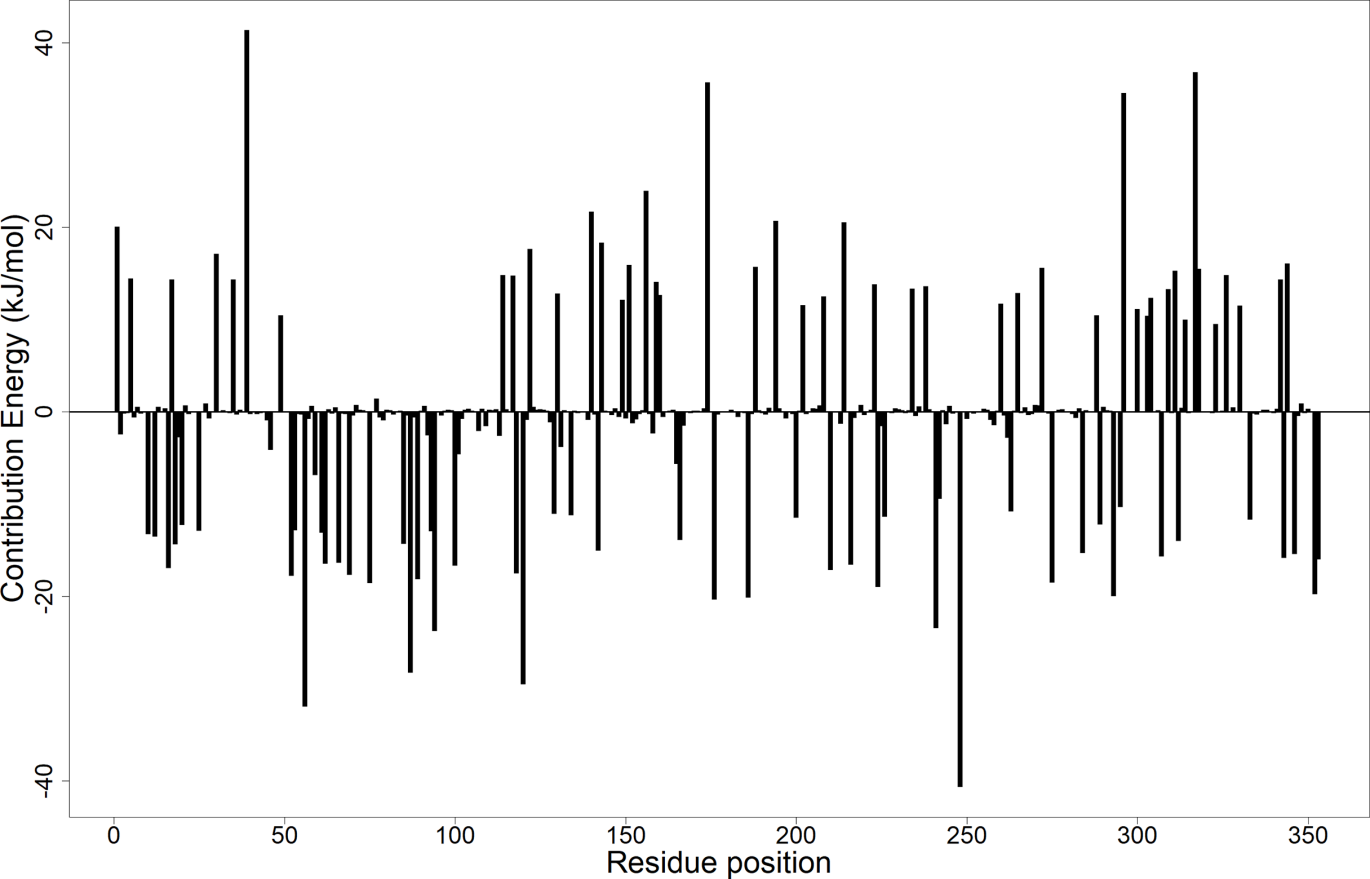
Molecular mechanics Poisson–Boltzmann surface area (MM-PBSA) plot showing the binding free energy contribution per residue of the *Ld*SMT–A1 complex.

Calculation of the energy contribution of each amino acid residue *via* per-residue energy analysis was performed using MM-PBSA (Congreve and Marshall, 2010; Grinter and Zou, 2014). It has previously been suggested that for a residue to contribute to the binding, the threshold must be >5 or <5 kJ/mol (Kwofie et al., 2019). Based on that, a detailed analysis of each complex was done (**Figure 7** and **Supplementary Figures 6A–C**), and for all the complexes, several binding site residues contributed favorable energies for ligand binding. The Tyr92 and Ala96 contributed to strong binding *via pi*–*pi* stacked, *pi*–*pi* T-shaped, *pi*–alkyl, and van der Waals interactions, while Asp58 through its hydrogen bonding strengthened the affinity for ligands bound in pocket 5.

### 3.13 Exploring the Antileishmanial Potential of the Predicted Compounds

**Figure 8:**
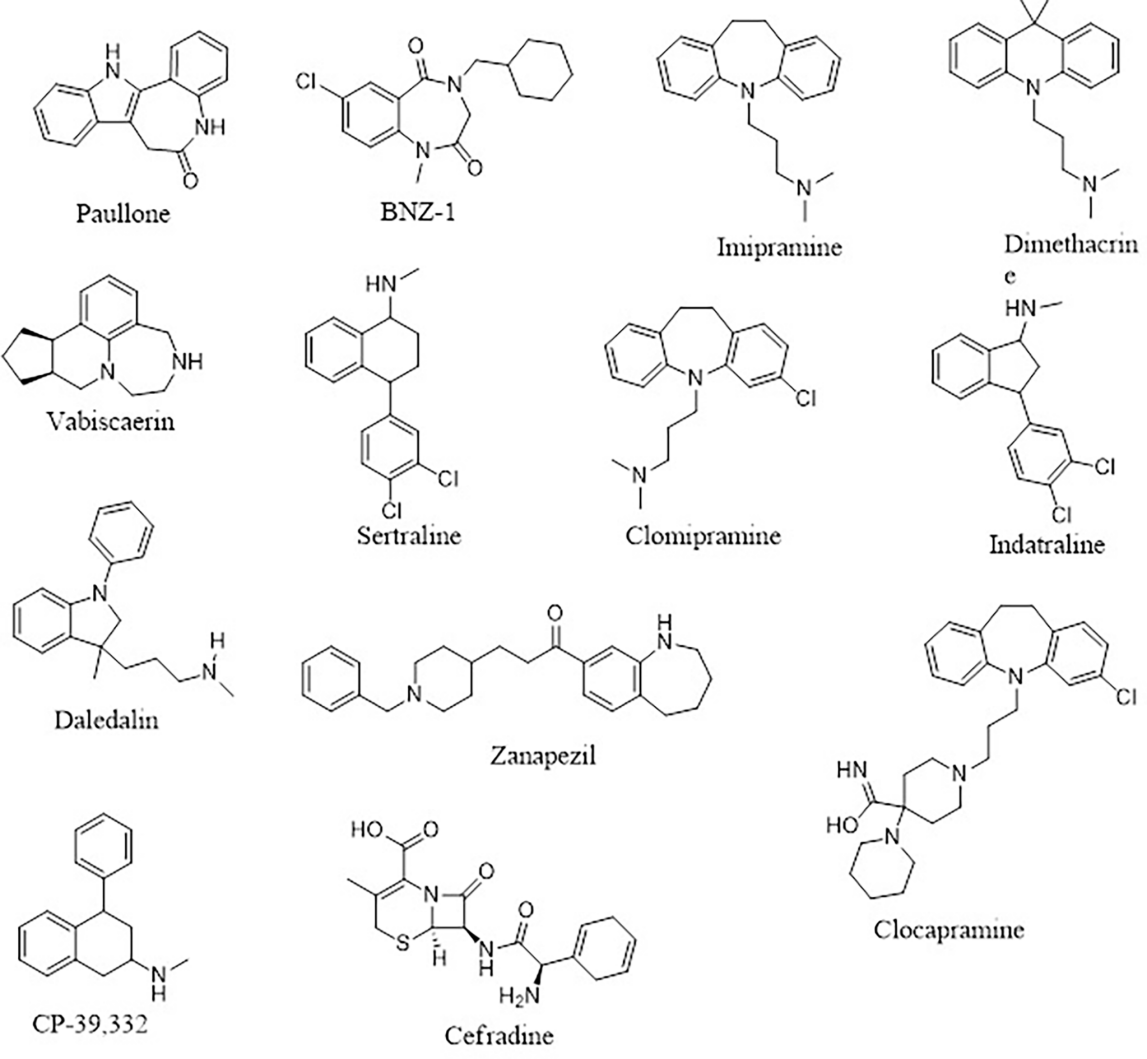
The 2D representations of the compounds cited from DrugBank.

Four (**A1**, **A2**, **A3**, and **A6**) out of the six compounds possessed benzo[*b*]azepine moiety as a replacement for the steroidal core in the 22,26-azasterol. This same moiety is present in paullone and its derivatives as well as BNZ-1 (**Figure 8**), which are known to suppress growth in *Leishmania* parasites with IC_50_ values of 47 nM and 100 μM, respectively (Clark et al., 2007; Dao Duong Thi et al., 2009), implying the possible antileishmanial potentials of the proposed hits. Moreover, the chemical structural similarity search of compounds **A1**, **A2**, **A3**, and **A6** *via* DrugBank (Wishart et al., 2018) revealed a variable similarity to antipsychotic and antidepressant drugs (Kaur, 2013; Mendonca Junior et al., 2015). For instance, compounds **A1**, **A2**, **A3**, and **A6** showed similarity scores above 0.50 to vabicaserin, sertraline, and indatraline (Richardson et al., 2009; Wishart et al., 2018; Lima et al., 2018). In addition, the four compounds showed similarity scores of around 0.55, which are close to those of daledalin, zanapezil, clocapramine, imipramine, and dimethacrine (**Figure 8**) (Mukherjee et al., 2012; da Silva Rodrigues et al., 2019). Interestingly, these drugs have been explored for their antileishmanial potentials causing *Leishmania* parasites to undergo mitochondrion depolarization in addition to inhibiting trypanothione reductase, thereby inducing strong oxidative stress in the parasite (Kaur, 2013; Andrade-Neto et al., 2016; da Silva Rodrigues et al., 2019). The similarity scores and the antileishmanial properties of these antidepressant and antipsychotic drugs warrant the testing of the compounds to assess their antileishmanial propensity.

Furthermore, compound **A4** devoid of the benzo[H]azepine also showed a chemical structural similarity score of 0.564 to CP-39,332, a serotonin–norepinephrine reuptake inhibitor. The successes emanating from other serotonin inhibitors for leishmaniasis treatment suggest **A4** as a potential antileishmanial compound. In addition, **A5** showed a similarity score of 0.538 to cefradine, a broad-spectrum antibiotic for the treatment of skin, chest, throat, and ear infections (Wishart et al., 2018). A recent study has revealed that patients exposed to antibiotics had a greater healing rate (Barakat et al., 2017), suggesting **A5** to be explored as an antileishmanial agent. In lieu of the aforementioned, the potential leads **A1**, **A2**, **A3**, **A4**, **A5**, and **A6** with diverse structural similarities with the antipsychotic and antibiotic agents are also proposed as potential antileishmanial agents *via* inhibition of sterol methyltransferase and are worthy of further experimental evaluation to assess their biological efficacy.

## 4 Potential Implications of the Study on *Leishmania donovani* Sterol Methyltransferase and Future Perspective

The study modeled a reasonable structure of *Ld*SMT with good quality parameters, which has been made available to augment the structure-based drug design. In addition, small non-steroidal molecules with negligible toxicity with the potential to suppress *Ld*SMT were identified and could be harmonized into non-commercial databases for the design of new biotherapeutic compounds. Furthermore, the *de-novo* design was employed in making available chemical structures of compounds which can be synthesized to ascertain their antileishmanial potency.

The renewed interest in polypharmacology drugs with the added advantage of overcoming drug resistance necessitates the investigation of these compounds against plausible targets involved in the ergosterol biosynthetic pathway of *Leishmania* parasites. Furthermore, coordinating these ligands to transition metals to find multimodality metallodrugs with the potential of inhibiting two or more enzymes in the ergosterol pathway may present a possible biotherapeutic route for leishmaniasis.

## 5 Conclusion

*In-silico* approaches were used to predict putative inhibitors targeting *Ld*SMT by elucidating the 3D structure of *Ld*SMT *via* Modeller followed by subjection of 22,26-azasterol to scaffold hopping and *de-novo* drug design. In all, six potential inhibitors labeled **A1**, **A2**, **A3**, **A4**, **A5**, and **A6** were generated *via de-novo* design with binding affinities of −8.4, −7.5, −7.2, −7.0, −7.0, and −7.0 kcal/mol, respectively. The compounds **A1** and **A2** demonstrated comparable binding affinity to that of 22,26-azasterol (−7.6 kcal/mol), the main inhibitor of *Ld*SMT. The study identified Tyr92 to be essential for ligand binding in the receptor binding pocket, and this was corroborated by MD simulation and MM-PBSA calculations. The physicochemical and pharmacological profiling showed that the compounds are drug-like and predicted as non-toxic. The predicted ligand quality metrics including *K_i_*, LE, LE_Scale, FQ, LELP, BEI, and SEI were all within the acceptable range. These findings suggest that the compounds possess antileishmanial potential and warrant experimental corroboration.

## Data Availability Statement

The original contributions presented in the study are included in the article/**Supplementary Material**. Further inquiries can be directed to the corresponding author.

## Author Contributions

POS, SKK, and RA conceptualized the project. PS designed the project and predominantly undertook the computational analysis with inputs from SKK, RA, EB, WAM, and MDW. POS wrote the first draft of the manuscript. All the authors read, edited, and approved the manuscript before submission.

## Conflict of Interest

The authors declare that the research was conducted in the absence of any commercial or financial relationships that could be construed as a potential conflict of interest.

## Publisher’s Note

All claims expressed in this article are solely those of the authors and do not necessarily represent those of their affiliated organizations, or those of the publisher, the editors and the reviewers. Any product that may be evaluated in this article, or claim that may be made by its manufacturer, is not guaranteed or endorsed by the publisher.
