# Supplementary figures and images for: Homology Modeling, de Novo Design of Ligands, and Molecular Docking Identify Potential Inhibitors of Leishmania donovani 24-Sterol Methyltransferase
Source: Front Cell Infect Microbiol. 2022 Jun 2;12:859981. doi: 10.3389/fcimb.2022.859981 (PMC9201040; doi:10.3389/fcimb.2022.859981)

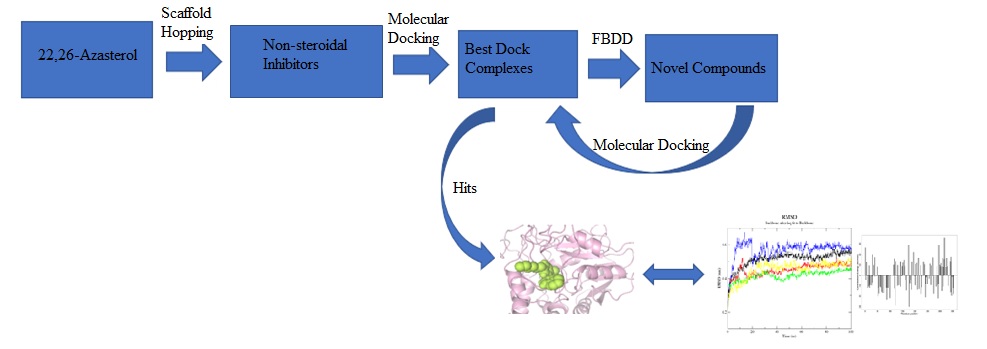

Supplement: Supplementary file 1 [file Image_1.jpeg]
