# Supplementary material for: Homology Modeling, de Novo Design of Ligands, and Molecular Docking Identify Potential Inhibitors of Leishmania donovani 24-Sterol Methyltransferase
Source: Front Cell Infect Microbiol. 2022 Jun 2;12:859981. doi: 10.3389/fcimb.2022.859981 (PMC9201040; doi:10.3389/fcimb.2022.859981)
Supplement: Supplementary file 2 [file Table_1.docx]

Supplementary Material

Homology Modeling, *De Novo* Design of Ligands and Molecular Docking Identify Potential Inhibitors of *Leishmania donovani* 24-Sterol Methyltransferase

Patrick O. Sakyi^1,2^, Emmanuel Broni^3,4^, Richard K. Amewu^1^, Whelton A. Miller III^5,6,7^, Samuel K. Kwofie^3,8*^

^1^Department of Chemistry, School of Physical and Mathematical Sciences, College of Basic and Applied Sciences, University of Ghana, P. O. BOX LG 56, Legon, Accra, Ghana.

^2^Department of Chemical Sciences, School of Sciences, University of Energy and Natural Resources, Box 214, Sunyani, Ghana.

^3^Department of Biomedical Engineering, School of Engineering Sciences, College of Basic & Applied Sciences, University of Ghana, Legon, Accra LG 77, Ghana.

^4^Department of Parasitology, Noguchi Memorial Institute for Medical Research (NMIMR), College of Health Sciences (CHS), University of Ghana, Legon, Accra LG 581, Ghana.

^5^Department of Medicine, Loyola University Medical Center, Maywood, IL, 60153, USA.

^6^Department of Molecular Pharmacology and Neuroscience, Loyola University Medical Center, Maywood, IL, 60153, USA.

^7^Department of Chemical and Biomolecular Engineering, School of Engineering and Applied Science, University of Pennsylvania, Philadelphia, PA, 19104, USA.

^8^Department of Biochemistry, Cell and Molecular Biology, West African Centre for Cell Biology of Infectious Pathogens, College of Basic and Applied Sciences, University of Ghana, P.O. Box LG 54, Accra, Ghana.

*** Correspondence:**
Dr. Samuel Kojo Kwofie
[skkwofie@ug.edu.gh](mailto:skkwofie@ug.edu.gh); Tel.: +233-203-737922

# Supplementary Tables

Supplementary Table 1: BLAST search results showing identical proteins to the *Ld*SMT. The best template is selected based on the E-value, sequence identity, BLAST score, and the resolution of the 3D structure. For all the 12 proteins, chain A was predicted as being identical to the *Ld*SMT.

| **PDB ID** | **Description** | **Max Score** | **Total Score** | **Query Cover** | **E-value** | **Percentage Identity** | **Accession Length** | **Resolution (Å)** |
| --- | --- | --- | --- | --- | --- | --- | --- | --- |
| 3BUS | Crystal Structure of RebM [*Lentzea aerocolonigenes*] | 89.4 | 89.4 | 63% | 6e-20 | 24.12% | 273 | 2.65 |
| 6UV6 | Chain A, D-glucose O-methyltransferase [*Actinomadura melliaura*] | 77.0 | 77.0 | 67% | 1e-15 | 23.20% | 268 | 2.72 |
| 5GM1 | Crystal structure of methyltransferase TleD complexed with SAH [*Streptomyces blastmyceticus*] | 75.9 | 75.9 | 48% | 5e-15 | 27.47% | 297 | 2.501 |
| 4PNE | Crystal Structure of the [4+2]-Cyclase SpnF [*Saccharopolyspora spinosa*] | 72.0 | 72.0 | 61% | 1e-13 | 24.15% | 302 | 1.50 |
| 5WP4 | Arabidopsis thaliana phosphoethanolamine N-methyltransferase 1 (AtPMT1, XIOPTL) in complex with SAH and phosphocholine [*Arabidopsis thaliana*] | 66.6 | 66.6 | 45% | 1e-11 | 29.01% | 491 | 1.341 |
| 5WP5 | Arabidopsis thaliana phosphoethanolamine N-methyltransferase 2 (AtPMT2) in complex with SAH [*Arabidopsis thaliana*] | 63.2 | 63.2 | 48% | 2e-10 | 26.90 | 491 | 1.5 |
| 4INE | Crystal structure of N-methyl transferase (PMT-2) from Caenorhabditis elegant complexed with S-adenosyl homocysteine and phosphoethanolamine [*Caenorhabditis elegans*] | 57.0 | 57.0 | 41% | 2e-08 | 28.38% | 454 | 1.45 |
| 4KRI | Haemonchus contortus Phospholethanolamine N-methyltransferase 2 in complex with phosphomonomethylethanolamine and S-adenosylhomocysteine [*Haemonchus contortus*] | 54.3 | 54.3 | 37% | 1e-07 | 25.74% | 433 | 1.72 |
| 4KRH | SeMet Haemonchus contortus Phosphoethanolamine N-methyltransferase 2 in complex with S-adenosyl-L-methionine [*Haemonchus contortus*] | 53.5 | 53.5 | 37% | 2e-07 | 25.74% | 433 | 3 |
| 6UAK | LahSb - C-terminal methyltransferase involved in RiPP biosynthesis [*Lachnospiraceae* bacterium C6A11] | 42.7 | 42.7 | 32% | 5e-04 | 27.35% | 308 | 2.01 |
| 4F84 | Structure analysis of Geranyl diphosphate methyltransferase in complex with SAM [*Streptomyces lasalocidi*] | 40.8 | 40.8 | 49% | 0.002 | 21.79% | 320 | 2.2 |
| 5EVJ | X-ray crystal structure of CrArsM, an arsenic (III) S-adenosylmethionine methyltransferase from Chlamydomonas reinhardtii [*Chlamydomonas reinhardtii*] | 38.1 | 38.1 | 43% | 0.020 | 25.00% | 387 | 2.4 |

Supplementary Table 2: Discrete optimized protein energy (DOPE), molpdf and GA341 scores of the generated models using Modeller 10.2.

| **MODEL** | **MOLPDF** | **DOPE SCORE** | **GA341 SCORE** |
| --- | --- | --- | --- |
| **3BUS-BASED MODELS** | | | |
| MOD3BUS1 | 2317.68604 | -29784.16797 | 0.99972 |
| MOD3BUS2 | 2363.51392 | -30234.79297 | 0.93211 |
| MOD3BUS3 | 2162.89307 | -29687.24219 | 0.96935 |
| MOD3BUS4 | 2203.05835 | -29973.73828 | 0.87870 |
| MOD3BUS5 | 2755.79858 | -29206.90820 | 0.99523 |
| **4PNE-BASED MODELS** | | | |
| MOD4PNE1 | 2310.66675 | -31158.07812 | 0.94163 |
| MOD4PNE2 | 3177.27271 | -30700.51172 | 0.73888 |
| MOD4PNE3 | 2313.64062 | -31110.76758 | 0.98537 |
| MOD4PNE4 | 2746.30908 | -31536.32422 | 0.98762 |
| MOD4PNE5 | 2255.64697 | -31608.05664 | 0.87840 |
| **3 TEMPLATES-BASED MODELS** | | | |
| MOD3TEMP1 | 13068.60742 | -31084.96094 | 0.94270 |
| MOD3TEMP2 | 12913.41113 | -30255.79688 | 0.64307 |
| MOD3TEMP3 | 13144.86230 | -31128.18359 | 0.85477 |
| MOD3TEMP4 | 12971.11523 | -31039.04492 | 0.91632 |
| MOD3TEMP5 | 12926.27441 | -30704.79102 | 0.70984 |

**Supplementary Table 3:** Model evaluation of the top 3 *Ld*SMT structures modelled via Modeller. E: error; W: warning; and P: pass.

| **Tool** | **Model Score** | | |
| --- | --- | --- | --- |
|  | **MOD3BUS2** | **MOD4PNE5** | **MOD3TEMP3** |
| Verify | 62.61 | 62.04 | 53.82 |
| Errat (Quality Factor) | 41.5663 | 46.9565 | 45.858 |
| Prove (%) | 11 (Error) | 7.1 (Error) | 9.6 (Error) |
| Procheck | 4 E, 1 W and 3 P | 4 E, 2 W and 2 P | 5 E, 1W and 2 P |

Supplementary Table 4: Predicted binding sites of the LdSMT and their respective area and volume obtained via CASTp. SA: Solvent accessibility.

| **Pocket** | **Area (SA)** | **Volume (SA)** | **Residues lining the pocket** |
| --- | --- | --- | --- |
| 1 | 541.956 | 185.351 | Thr11, Asn12, Asn23, Val26, Ala29, Ala30, Phe33, Arg34, Arg36, Phe37, Thr51, Met52, Val53, Asn54, Phe83, Leu87, Asp108, Val109, Gly110, Cys111, Gly112, Val113, Pro116, Val131, Asn132, Asn133, Gln137, Thr159, Asp160, Phe161, Cys162, Ala176, Ile177, Glu178, Ala179, Cys181, His182, Ala183, Lys186, Cys189, Tyr190, Val193, Phe203, Trp208, Arg222, Thr223, Ile224, Lys225, Trp274, Tyr275, Leu278, Tyr282, Lys317, Ala318, Val321 |
| 2 | 142.370 | 160.914 | Asp31, Arg32, Arg34, Asp35, Arg36, Lys39,Tyr136, Met301, Val304, Glu306, Phe307, Val308, Arg309, Leu310, Ala311 |
| 3 | 64.974 | 138.850 | Trp68, Gly69, Gln70, Asn71, Tyr78, Gln276, Pro277, Val279, Gly280, Ser283, Leu288, Arg289 |
| 4 | 85.790 | 39.651 | Leu13, Ile14, Arg15, Pro216, Asp218, Glu219, Tyr220, His221, Thr223, Tyr316, Glu320, Leu329 |
| 5 | 76.459 | 35.252 | Gly98, Gly99, Phe100, Met101, Asp104, Ile106, Asp172, Gly173, Ala174, Tyr175, Gly200, Thr201, Cys202, Val204, Ile344 |
| 6 | 64.780 | 28.523 | Glu43, Arg45, Thr49, Thr50, Thr51, Phe84, Gly115, Arg118, Asn119, Arg122, His144 |
| 7 | 83.722 | 20.376 | Glu91, Tyr92, Ala95, Gly99, Met101, Asp104, Ile106, Asn119, Met120, Leu123, Thr124, Cys126, Val128, Tyr175 |

Supplementary Table 5: Scores of superimposed compounds identified by scaffold hoping.

| **Compound ID** | **Superimposed Structure** | **Total Score** | **LiSiCA** | **WEGA** | **Optipharm** | **Screen3D** |
| --- | --- | --- | --- | --- | --- | --- |
| CHEMBL120903 | 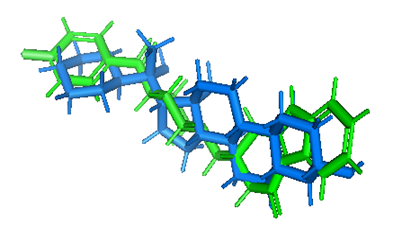 | 0.65085 | 0.59460 | 0.66960 | 0.66919 | 0.67000 |
| CHEMBL1079072 | 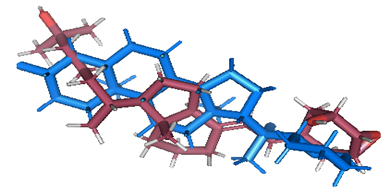 | 0.64871 | 0.61111 | 0.67714 | 0.67658 | 0.63000 |
| CHEMBL101625 | 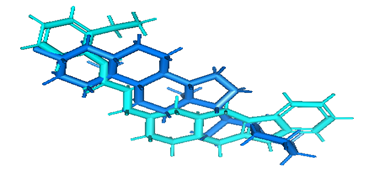 | 0.59318 | 0.51282 | 0.59659 | 0.59331 | 0.67000 |
| CHEMBL463489 | 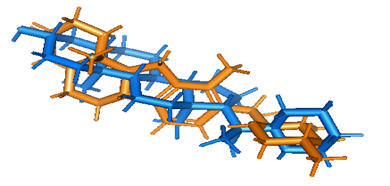 | 0.56786 | 0.51282 | 0.66930 | 0.66931 | 0.42000 |
| CHEMBL453882 | 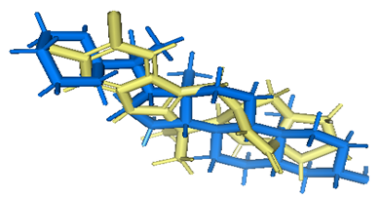 | 0.51089 | 0.30233 | 0.69604 | 0.69520 | 0.35000 |
| CHEMBL66430 | 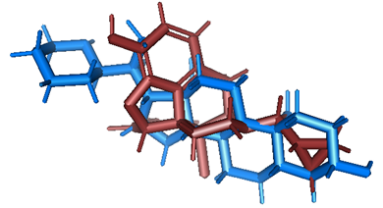 | 0.44799 | 0.28571 | 0.57380 | 0.57244 | 0.36000 |
| CHEMBL230669 | 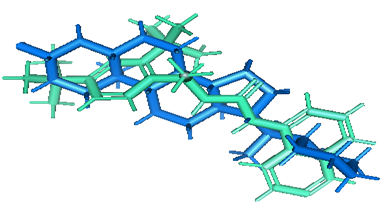 | 0.41643 | 0.20408 | 0.61048 | 0.59115 | 0.26000 |
| CHEMBL400343 | 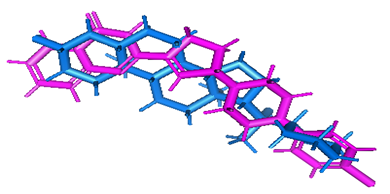 | 0.40832 | 0.1600 | 0.63956 | 0.63372 | 0.20000 |
| CHEMBL349416 | 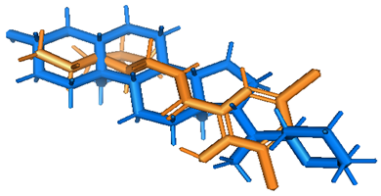 | 0.40558 | 0.19512 | 0.59380 | 0.59341 | 0.24000 |
| CHEMBL434964 | 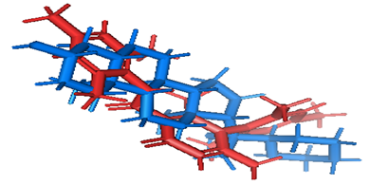 | 0.39841 | 0.15686 | 0.66535 | 0.66141 | 0.11000 |

Supplementary Table 6: The binding affinities and intermolecular interactions between *Ld*SMT and predicted scaffolds, known inhibitors and drugs.

| **Compounds** | **Binding Affinity (kcal/mol)** | **Interacting residues** | |
| --- | --- | --- | --- |
|  |  | **Hydrogen Bonds** | **Hydrophobic Bonds** |
| **Known Inhibitors** | | | |
|  | -7.6 | Glu102, Gly200 | Phe100, Lys198, Pro199 |
|  | -7.3 | Asp172, Gly200 | Cys202 |
|  | -7.0 | - | Phe100, Gly200, Cys202, Tyr343, Ile344 |
|  | -6.2 | Asp172 | Phe100, Lys198, Pro199, Gly200, Ile344 |
|  | -6.5 | Gly200 | Phe100, Asp172, Lys198, Ile344 |
|  | -7.7 | Asn12, Thr319 | Lys313, Tyr316 |
|  | -5.9 | - | Ala95, Ala96, Met120, Thr124 |
|  | -7.2 | - | Glu44, Phe84, Ala88, Tyr92 |
| **Drugs** | | | |
|  | -5.3 | Asp31, Phe307, Val308, Arg309, Leu310 | Asp35 |
|  | -5.0 | Arg89 | - |
|  | -4.0 | Cys202 | Phe100, Met101, Asp104, Asp172, Pro199, Gly200, Thr201, Tyr343, Ile344 |
| **12 compounds from scaffold hopping** | | | |
|  | -9.0 | - | Asp58, Ala88, Arg89, Tyr92, Phe264 |
|  | -8.9 | Arg89 | Asp58, Ala88, Tyr92 |
|  | -8.8 | Arg89 | Asp58, Ala88, Arg89, Tyr92, Phe264 |
|  | -8.7 | Arg89 | Asp58, Ala88, Arg89, Tyr92, Phe264 |
|  | -7.7 | - | Asp58, Arg89, Phe93, Phe264 |
|  | -7.6 | Asp58 | Arg89, Tyr92, Ala95, Ala96, Leu123 |
|  | -7.4 | Asp58 | Ala88, Arg89, Tyr92, Phe93 |
|  | -7.4 | Gly287 | Leu288, Arg295, Leu322 |
|  | -7.3 | - | Arg89, Tyr92, Ala96, Phe93 |
|  | -7.2 | - | Arg89, Tyr92, Ala95, Ala96, Phe93 |
|  | -7.0 | Arg89 | Ala88, Tyr92, Phe264 |
|  | -7.0 | - | Asp58, Ala88, Arg89, Tyr92, Phe93, Phe264 |

Supplementary Table 7: Predicted physicochemical properties of *de novo* designed compounds. Molecular weight (M. W), hydrogen bond acceptor (HBA), hydrogen bond donor (HBD), gastrointestinal absorption (GI), blood brain barrier (BBB), number of Lipinski’s rule violation (L.P.V), number of Veber’s rule violation (V.V), synthetic accessibility (SA) and lead likeness violation (L.V) are reported.

| **Ligand** | **M.W** | **HBA** | **HBD** | **TPSA** | **clogP** | **ESOL Class** | **GI** | **BBB** | **Pgp Substrate** | **L.P.V** | **V.V** | **S.A** | **L.V** |
| --- | --- | --- | --- | --- | --- | --- | --- | --- | --- | --- | --- | --- | --- |
| A1 | 393.95 | 2 | 0 | 29.02 | 5.62 | Poorly soluble | High | No | Yes | 1 | 0 | 3.9 | 2 |
| A2 | 411.02 | 0 | 1 | 15.27 | 6.89 | Poorly soluble | Low | No | Yes | 1 | 0 | 4.13 | 2 |
| A3 | 315.54 | 0 | 1 | 12.03 | 6.33 | Poorly soluble | Low | No | Yes | 1 | 0 | 3.6 | 2 |
| A4 | 372.54 | 2 | 2 | 58.2 | 4.61 | Moderately soluble | High | Yes | No | 0 | 1 | 3.77 | 3 |
| A5 | 300.37 | 5 | 3 | 107.33 | -0.1 | Very soluble | High | No | Yes | 0 | 0 | 4.77 | 0 |
| A6 | 398.54 | 4 | 3 | 64.96 | 3.38 | Moderately soluble | High | Yes | Yes | 0 | 0 | 4.68 | 2 |

Supplementary Table 8: Toxicity prediction of the *de novo* compounds via Osiris Data Warrior

| **Ligand** | **Data Warrior Predictions** | | | |
| --- | --- | --- | --- | --- |
|  | **Tumorigenic** | **Mutagenic** | **Irritant** | **Reproductive Effect** |
| A1 | High | None | None | None |
| A2 | None | None | None | None |
| A3 | None | None | None | None |
| A4 | None | None | None | None |
| A5 | None | None | None | None |
| A6 | None | None | None | None |
| 22,26-azasterol | None | None | None | None |
| X1 | None | None | None | None |
| X2 | None | None | None | None |
| X3 | None | None | None | None |
| X4 | None | None | Low | None |
| X5 | None | None | None | None |
| X6 | None | None | None | High |
| X7 | None | None | None | None |
| Amphotericin B | None | None | None | None |
| Miltefosine | None | None | None | None |
| Paromomycin | None | None | None | None |

**Supplementary Table 9:** Binding energy and quality assessment metrics of predicted analogous.

| **Compound** | **B.E** | **Ki (μM)** | **LE** | **LE_Scale** | **FQ** | **LELP** | **BEI** | **SEI** |
| --- | --- | --- | --- | --- | --- | --- | --- | --- |
| **A1** | -8.4 | 0.695 | 0.300 | 0.358 | 0.838 | 18.733 | 15.631 | 21.220 |
| **A2** | -7.5 | 3.174 | 0.259 | 0.347 | 0.744 | 24.942 | 13.376 | 36.005 |
| **A3** | -7.2 | 5.268 | 0.313 | 0.416 | 0.752 | 20.224 | 16.727 | 43.874 |
| **A4** | -7.0 | 7.386 | 0.259 | 0.369 | 0.702 | 17.800 | 13.775 | 8.817 |
| **A5** | -7.0 | 7.386 | 0.350 | 0.455 | 0.769 | 5.371 | 17.105 | 4.781 |
| **A6** | -7.0 | 7.386 | 0.241 | 0.347 | 0.695 | 14.025 | 12.876 | 7.900 |
| 22,26-azasterol | -7.6 | 2.681 | 0.262 | 0.347 | 0.755 | 17.137 | 13.804 | 10.615 |

# Supplementary Figures

# A)

#
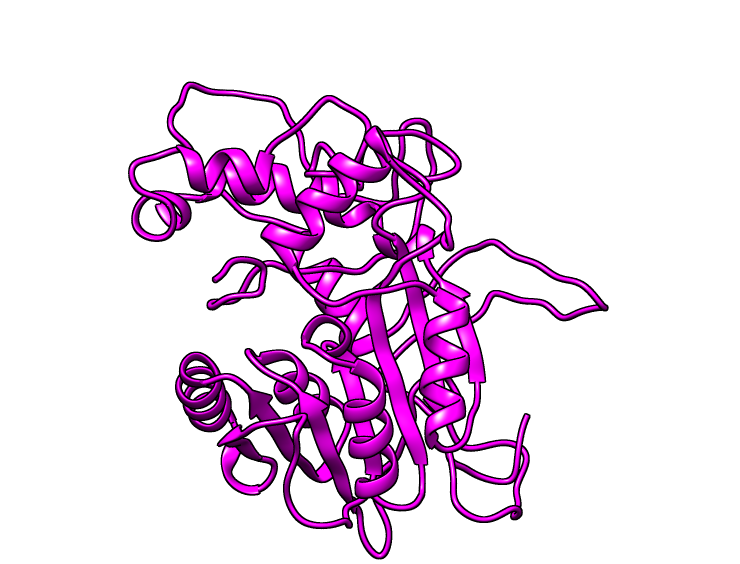


**B)**


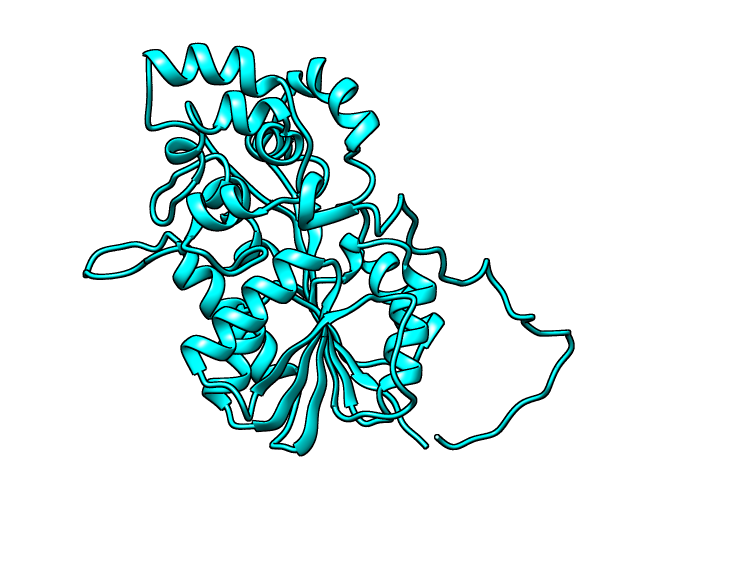


**C)**


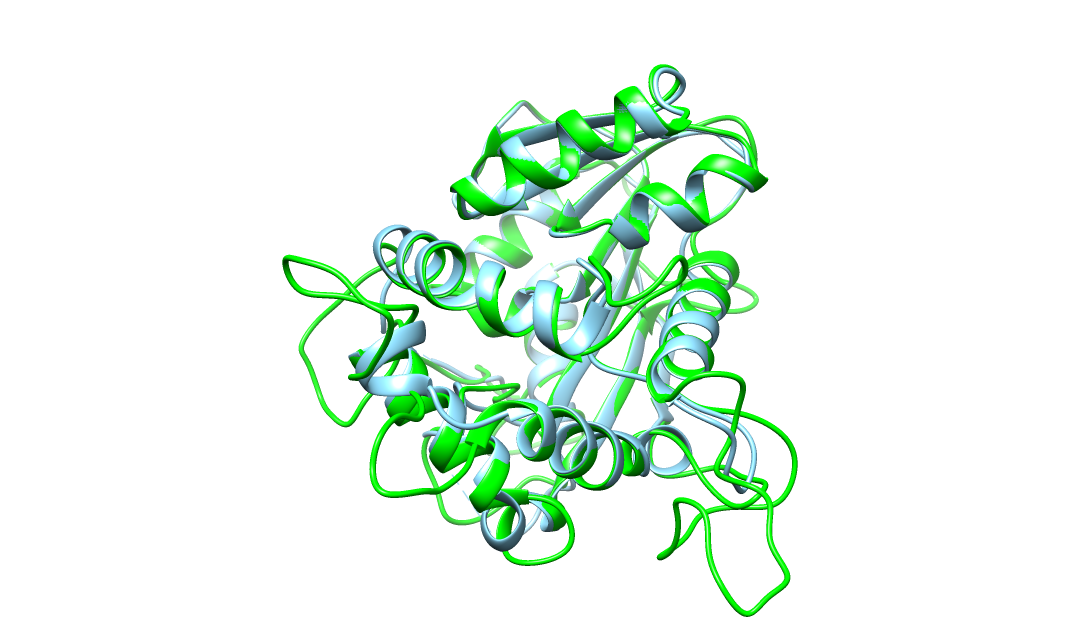


**Supplementary Figure 2.** Cartoon representation of the structures of the (**A**) MOD3BUS2, (**B**) MOD3TEMP3, and (**C**) alignment of MOD4PNE5 and the 4PNE template. For C), MOD4PNE5 and 4PNE are colored green and cyan, respectively.

**A)**


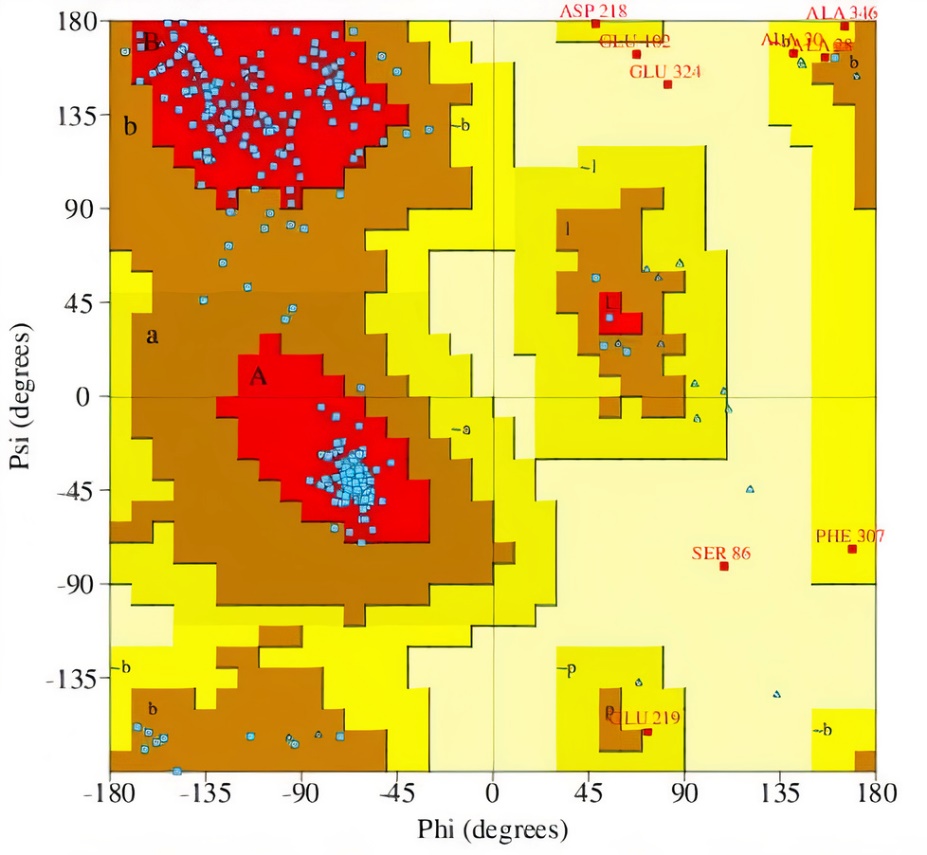


**B)**


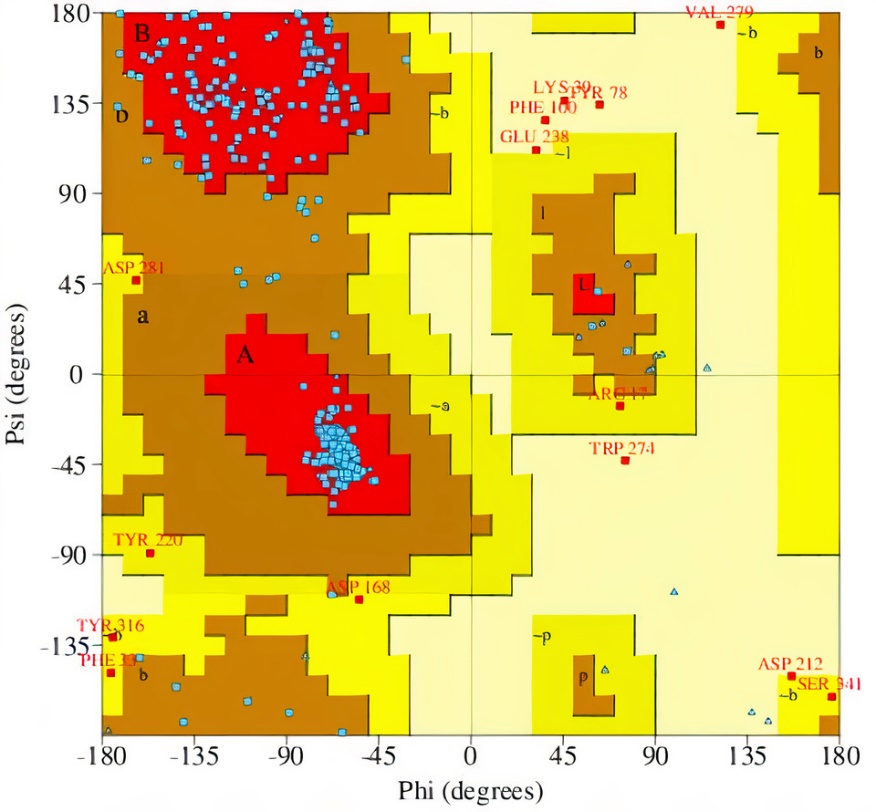


**Supplementary Figure 3.** Ramachandran plot obtained via PROCHECK for (**A**) MOD3BUS2 and (**B**) MOD3TEMP3.

**A)**


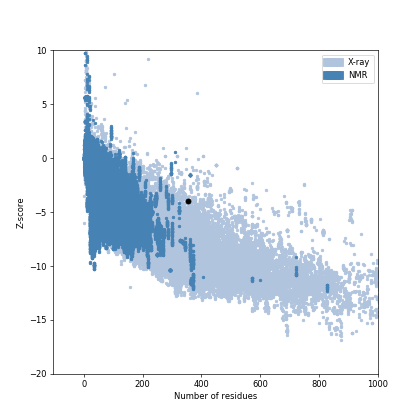


**B)**


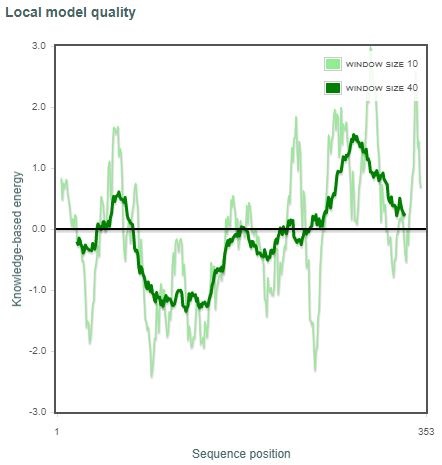


**Supplementary Figure 4.** Quality assessment analysis of the selected *Ld*SMT model from ProSA-web. (**A**) A z‐score indicating the quality of the 3D protein structure, and (**B**) local model quality of the *Ld*SMT structure.

**A)**


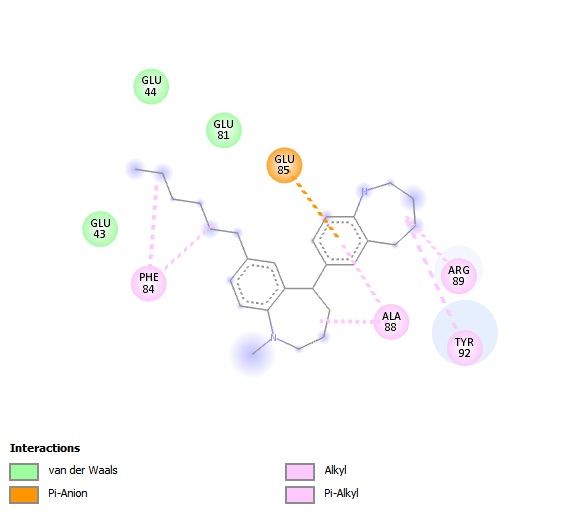


**B)**


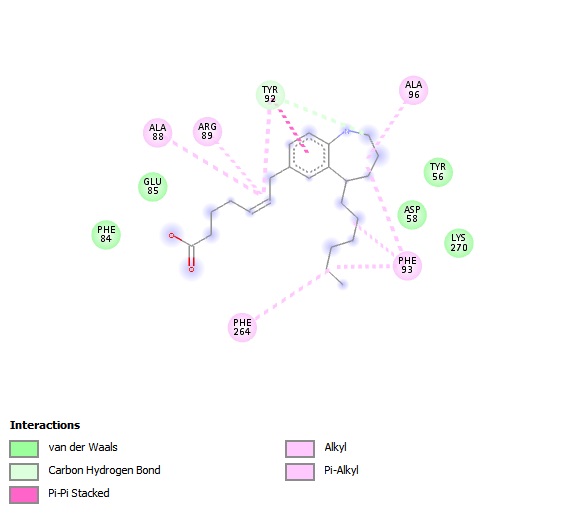


**C)**


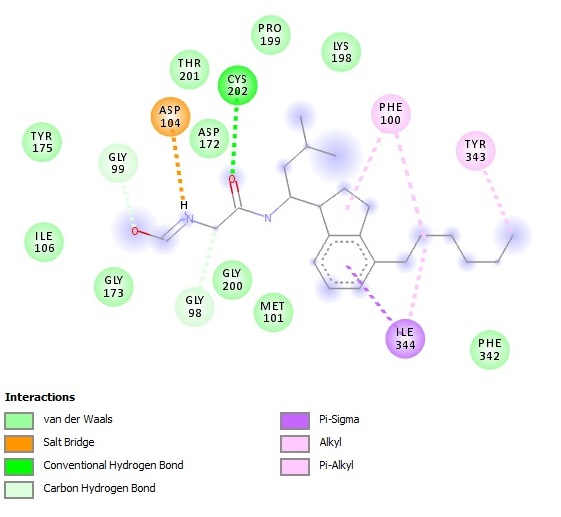


**D)**


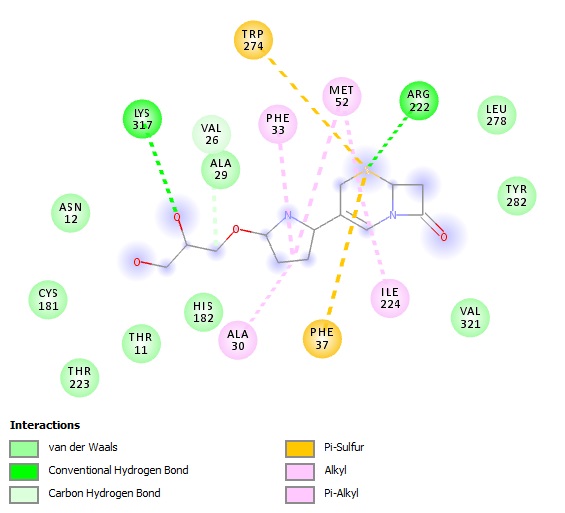


**E)**


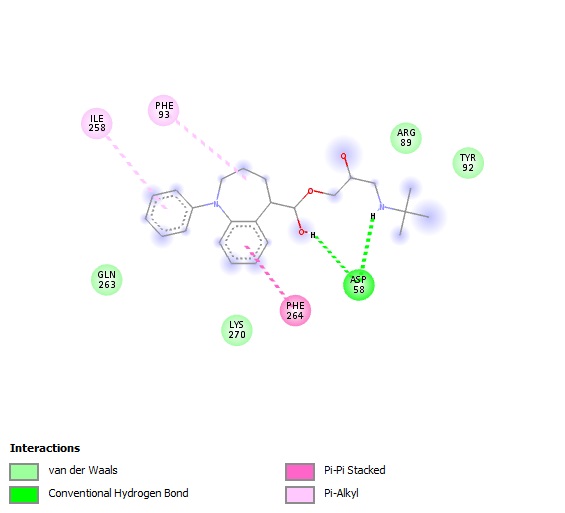


**Supplementary Figure 5.** 2D interaction profiles of the *Ld*SMT-*de novo* hit complexes as visualized in Discovery studio. The interaction profiles of *Ld*SMT- (**A**) **A2**, (**B**) **A3**, (**C**) **A4**, (**D**) **A5** and (**E**) **A6** complexes are presented.

**A)**


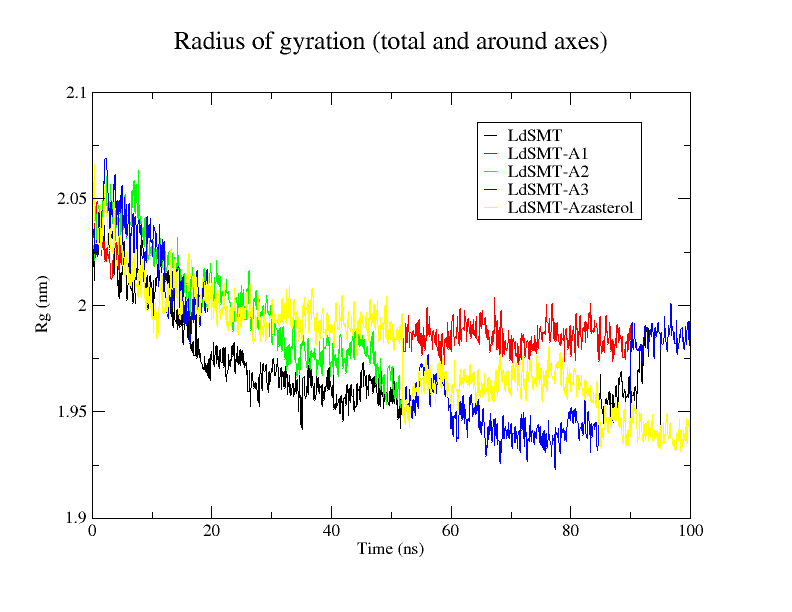


**B)**


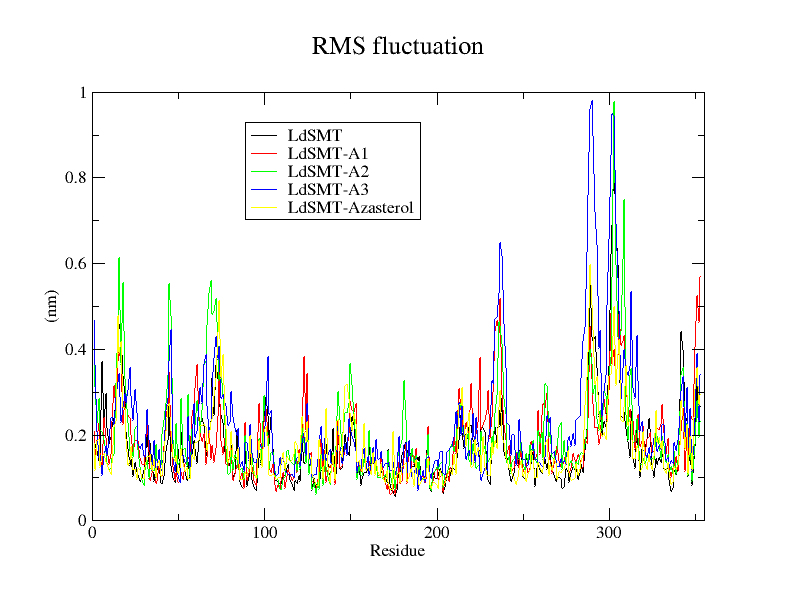


**Supplementary Figure 6.** Root mean square fluctuations (RMSF) and the radius of gyration (Rg) plots of 100 ns molecular dynamics (MD) simulations of the *Ld*SMT-ligand complexes using GROMACS. (**A**) Rg versus a time graph of the *Ld*SMT-ligand complexes, and (**B**) analysis of RMSF trajectories of residues of the *Ld*SMT-ligand complexes.

**A)**


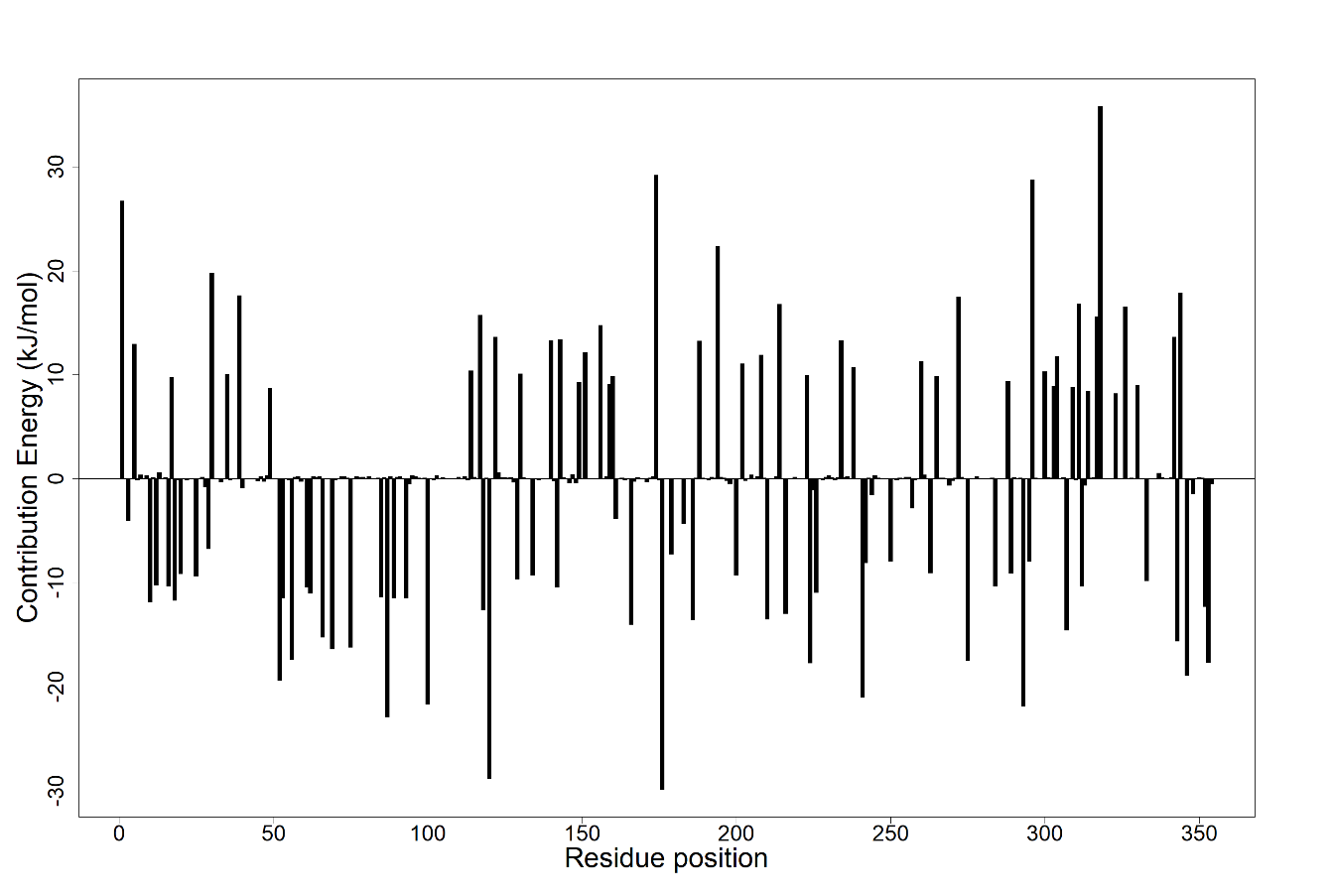


**B)**


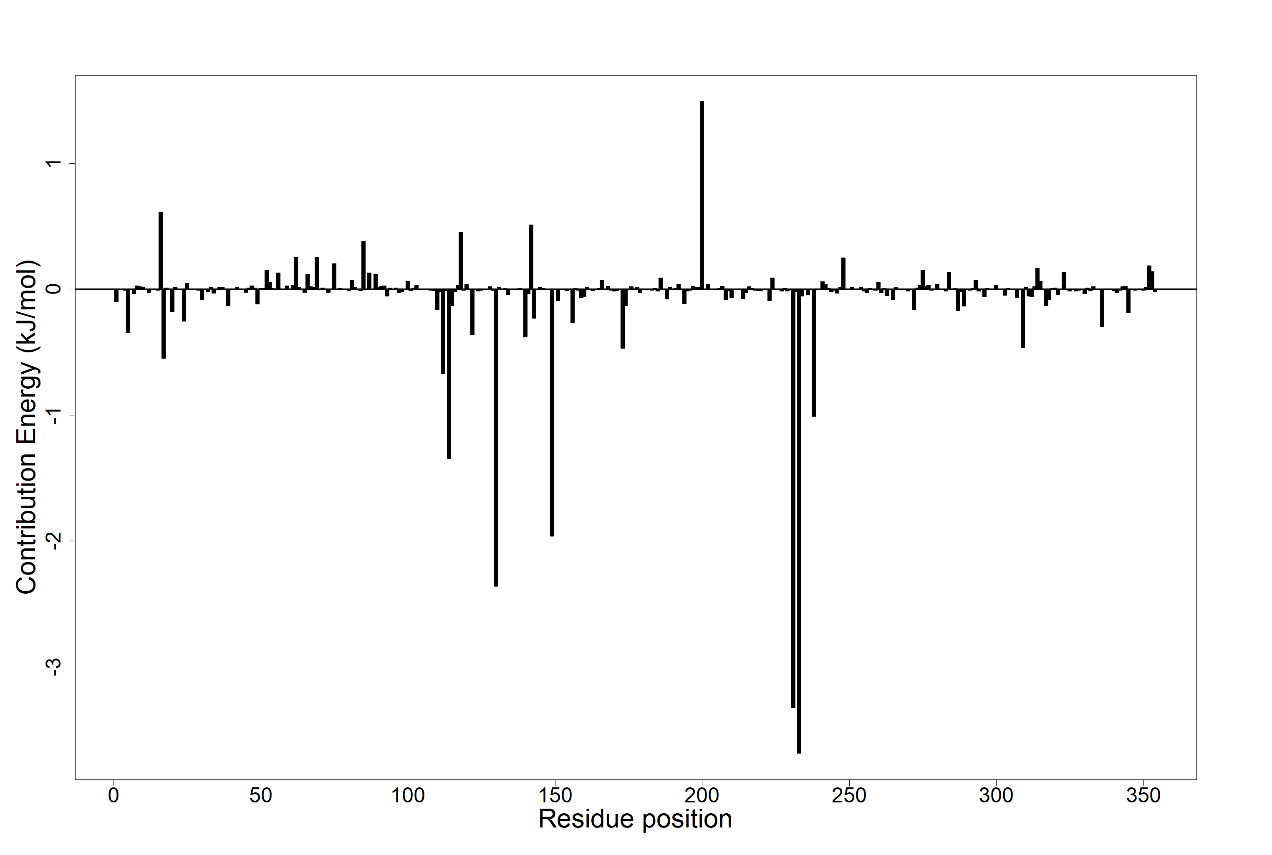


**C)**


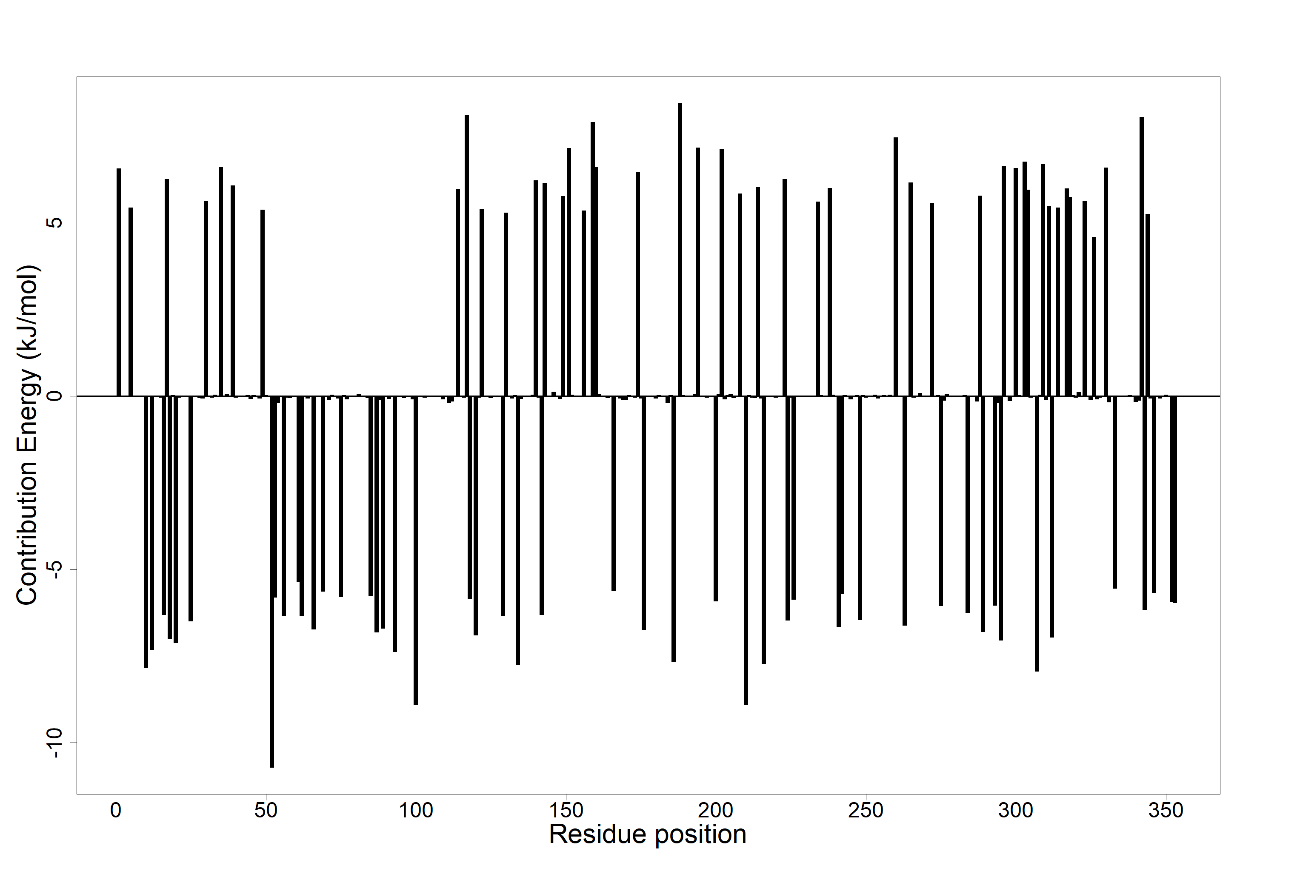


**Supplementary Figure 7.** Molecular mechanics/Poisson-Boltzmann surface area (MMPBSA) plot showing the binding free energy contribution per residue of the (**A**) *Ld*SMT-A2, (**B**) *Ld*SMT-A3, and (**C**) *Ld*SMT-azasterol complexes.
